# A Rapid Screening Method for the Detection of Additives in Electronics and Plastic Consumer Products Using AP-MALDI-qTOF-MS

**DOI:** 10.3390/toxics11020108

**Published:** 2023-01-23

**Authors:** Maurice de Jonker, Pim E. G. Leonards, Marja H. Lamoree, Sicco H. Brandsma

**Affiliations:** Amsterdam Institute for Life and Environment (A-LIFE), Section Environment and Health, Vrije Universiteit, Amsterdam, De Boelelaan 1085, 1081HV Amsterdam, The Netherlands

**Keywords:** AP-MALDI, plastic consumer products, plastic additives, suspect screening, recycling

## Abstract

A novel method was developed and optimized for the fast-screening analysis of additives in electronics and plastic consumer products using atmospheric pressure matrix-assisted laser desorption ionization (AP-MALDI) coupled with a high-resolution quadrupole time-of-flight (qTOF) mass spectrometer (MS). To simplify sample preparation and increase sample throughput, an innovative 48 well graphene nanoplatelets (GNP) doped AP-MALDI target plate was developed. The GNP incorporated in the target plate fulfilled the role of the MALDI matrix and, therefore, sample extracts could be directly transferred to the AP-MALDI 48 well target plate and analyzed without a subsequent matrix addition. The homogeneously dispersed and immobilized GNP target plates also provided increased signal intensity and reproducibility. Furthermore, analytical standards of various plastic additives and plastic products with known concentrations of additives were studied to assess the AP-MALDI ionization mechanisms and method capability. The analysis time was 15 s per measurement using an automated sequence. The GNP-doped target plates exhibited high desorption/ionization of low molecular weight molecules (<1000 Da) and can be used in both positive and negative ionization modes. The AP-MALDI-qTOF-MS method was applied to screen for additives in various electronics and plastic consumer products. Suspect screening was performed using a database containing 1366 compounds. A total of 56 additives including antioxidants, flame retardants, plasticizers, UV-stabilizers, and UV-filters were identified (confidence level 4). Identification of certain plastic additives in plastic children’s toys may indicate that they are recycled from waste electronic and electronic equipment (WEEE).

## 1. Introduction

Plastics are lightweight, relatively inexpensive, durable, and versatile materials that have become practically unavoidable in modern life. Pristine polymeric materials usually do not possess the desired manufacturing and end-use properties [1]. Plastics are therefore often modified using additives such as pigments, dyes, fillers, antioxidants, flame retardants, UV stabilizers, and plasticizers to achieve the desired properties. In addition to the intentionally added additives, other chemicals can be present in plastics, such as unreactive intermediates, monomers, impurities, and breakdown products [2]. Additives are generally not chemically bound to the polymer [1,2,3]. During the plastic’s lifetime, additives can migrate from plastic products, which may result in human and environmental exposure [4]. Bisphenol A (BPA), di(2-Ethylhexyl) phthalate (DEHP), dibutyl phthalate (DBP), and some brominated flame retardants (BFRs) are examples of additives that have been proven to be harmful to life after years of unrestricted use in household plastics [3,5,6]. Equally alarming is the lack of publicly available toxicity data on a large number of plastic additives [2,6,7]. Twelve phthalates are classified as substances of very high concern on the European Chemicals Agency (ECHA) candidate list [8], nine phthalates are on the ECHA authorization list and need approval to be used in specific products [9]. DEHP, DBP, and butyl benzyl phthalate (BBP) are not allowed in baby toys in concentrations above 0.1 wt.% by EU directives [10]. Diisononyl phthalate (DINP), diisodecyl phthalate (DIDP), and di(n-octyl) phthalate (DNOP) are not allowed in children’s toys that are intended to be placed in the mouth, in concentrations above 0.1 wt.% [10]. The usage of these five phthalates, DEHP, DBP, BBP, DINP, and DIDP, are also restricted in food contact plastics [11]. The U.S. Consumer Product Safety Commission (CPSC) banned the manufacturing and sale of children’s products containing more than 0.1 wt.% of eight phthalates [12]. The increasing complexity of regulations caused by the toxicity of these additives produces challenges in verifying regulatory compliances. With increasing interest in a circular economy, and therefore recycling, toxic additives have become an even larger concern [13,14]. Inappropriate recycling of plastics containing toxic additives may lead to more environmental and human exposure [4]. To produce safe products from recycled plastic, it is fundamental to separate the “good” from the “bad” recyclates. The wide variety of additives present in plastic products causes target analysis to be costly and time-consuming [15]. To control the quality and safety of plastic products, it is important to develop screening methods capable of quickly identifying a wide range of (potentially toxic) additives and contaminants in plastic products. Recently, the concept of screening plastics for the presence of additives has received significant attention. Direct atmospheric pressure mass spectrometry methods have been proposed as a suitable method [16]. Direct Analysis in Real-Time (DART) [17,18], desorption Electro Spray Ionization (DESI) [19], Atmospheric Solid Analysis Probe (ASAP) [20], and Direct Inlet Probe (DIP)-APCI [15] have been used to screen additives in polymers. Although these methods can be used to detect additives directly from plastics, for some of these methods, the absence of an autosampler, in-/near-source contamination, multiple optimization parameters, and lack of reproducibility hinders the high throughput potential of these methods. To address these shortcomings, the use of atmospheric pressure matrix-assisted laser desorption ionization (AP-MALDI) in combination with time-of-flight mass spectrometry (qTOF) was investigated and used to screen additives in plastics.

The main advantages of an AP-MALDI over a vacuum MALDI are the lower cost, the ability to analyze volatile compounds, the simplicity of sample introduction, and the possibility of coupling the source to various MS instruments [21,22,23,24]. (AP)-MALDI is a widely used soft ionization method to study biopolymers such as proteins and peptides [23,24]. It is usually performed on a stainless-steel target plate, whereby a matrix is used, that co-crystallizes with the analytes to absorb the laser energy and facilitate ionization for MS detection. Traditional organic matrices can ionize, fragment, and form adducts which reduce sensitivity and impede identification in the low molecular weight region (<500 Da) [25]. Recently, promising results have been demonstrated using inorganic matrices for the analysis of small molecules. The use of graphene as a MALDI matrix especially shows promising results [26,27,28,29,30,31,32,33,34,35,36]. Graphene provides MALDI-MS spectra without matrix peaks and matrix adducts, and demonstrates excellent salt tolerances [37,38]. Improved reproducibility could be achieved compared to organic matrices, due to the elimination of the need for co-crystallization [37,38]. However, graphene matrices are not without problems, e.g, mainly the aggregation of graphene, resulting in an inhomogeneous matrix layer on the plate reducing the reproducibility [27]. Wang et al. [27] used 3D-printing to fabricate graphene-doped MALDI plates for vacuum MALDI-MS, whereby photocurable resin was mixed with a small amount of graphene (0.1 wt.%) and the target plates were directly printed using a commercial desktop stereolithographic (SLA) 3D-printer. The 3D-printed graphene-doped MALDI target plates showed promising results while analyzing standard solutions of low molecular mass and environmental pollutants, as well as a synthetic polypeptide [27]. The 3D-printed target plates showed increased reproducibility compared to traditional MALDI methods due to the immobilization of the graphene.

In our study, we implemented the graphene-doped MALDI target plate as discussed by Wang et al. [27] and further modified it for an AP-MALDI application. The modifications in the sample plate design included dedicated wells, to limit cross-contamination, and accurate deposition of larger sample volumes. This study aimed to develop a fast-screening method using AP-MALDI-qTOF-MS to simultaneously screen a wide range of plastics for a broad selection of additives below 1000 mg L^−1^ (0.1 wt.%). To achieve this, graphene nanoplatelets (GNP) doped resin target plates were developed and validated for AP-MALDI analysis. The developed method was subsequently applied to screen for additives in various plastic consumer products.

## 2. Experimental Section

### 2.1. Materials and Reagents

GNP (grade C, surface area 750 m^2^ g ^−1^, particle size 2 μm, with a thickness of a few nm, relative gravity: 2.00–2.25 g (cm^3^)^−1^) were purchased from Sigma Aldrich. Epoxy resin (Resion EP101, EP121), unsaturated polyester (UP) resin (Albastine), polyurethane (PUR) resin (Axson F160), and two-part silicone rubber (Resion, SR1-15A and SR1-15B) were purchased from local stores in the Netherlands; more details are given in the Supplementary Materials. The 3D-printer used for printing the GNP-doped MALDI plates was a stereolithography (SLA) 3D-printer from Formlabs (Form 2) with Formlabs photopolymer clear resin (FLGPCL04; Somerville, MA, USA).

### 2.2. 3D-Printing of the GNP-Doped AP-MALDI Target Plates

A model of the AP-MALDI target plate (44 mm by 44 mm) was designed using Fusion 360 software (Autodesk Inc., San Francisco, CA, US) to fit the AP-MALDI target plate holder. The GNP-doped AP-MALDI targets were printed as described by Wang et al. [27], using 0.01 and 0.1 wt.% GNP. The GNPs were stirred into 3D-printing photocurable clear resin and the target plates were directly printed using a desktop stereolithography 3D-printer (Form 2, Formlabs). Detailed information is given in the Supplementary Materials.

### 2.3. Resin Casting of the GNP-Doped AP-MALDI Target Plates

A model of the AP-MALDI target plate was produced using 3D-printing (Figure S1). A mold of this model was made using two-part silicone rubber (Figure S2). The silicone mold was used for casting the GNP-doped epoxy resin, PUR resin, and UP resin AP-MALDI target plates (Figure S3). A detailed description of the casting procedure for the three synthetic resins using this silicone mold is given in the Supplementary Materials.

### 2.4. Preparation Standard Solutions

The performance of the GNP-doped AP-MALDI targets was tested with a standard mixture containing 80 mg L^−1^ tetrabromobisphenol A (TBBPA), 110 µg mL^−1^ triphenyl phosphate (TPhP), 150 µg mL^−1^ dibutyl phthalate (DBP), 140 mg L^−1^ tris(2,4-di-tert-butylphenyl)phosphite (Irgafos 168), 140 mg L^−1^ bis(2,2,6,6,-tetramethyl-4-piperidyl)sebacate (Tinuvin 770), and 140 mg L^−1^ 2,4,6-tris(2,4,6-tribromophenoxy)-1,3,5-triazine (TTBP-TAZ) in toluene. The observed molecular ions and their theoretical *m/z* values are summarised in Table S1. A variety of plastic additive standards were used to further evaluate the performance of the AP-MALDI method and assess the adduct formation (see Table S2).

### 2.5. Sample Preparation

Detailed information on the extraction optimization is given in the Supplementary Materials. To briefly explain, samples were cut into small pieces (<2 mm) using a disposable blade. Approximately 30 mg of plastic sample was transferred to a 2 mL LC vial with 200 µL insert, 20 µL of toluene was added to the samples to dissolve or swell the polymer followed by sonication for 10 min. Afterward, 80 µL of a saturated KCl in 2-propanol solution was added to extract and precipitate the polymer. The addition of the saturated KCl solution resulted in increased sensitivity through [M+K]^+^ adduct formation. The samples were sonicated for an additional 10 min and centrifuged for 10 min at 5000 rpm. For each sample, 2 µL of the supernatant was pipetted in triplicate into the wells of the GNP-doped UP AP-MALDI plate. The plates were dried in a fume hood at room temperature and analyzed with the AP-MALDI-qTOF-MS. Each GNP-doped AP-MALDI plate contained three blanks and a perfluoroalkane sulfonic acid (PFSA) calibration mix in the first and last well to calibrate the qTOF, to determine the reproducibility between the target plates. Each GNP-doped AP-MALDI plate contains 48 wells (Figure S3). The total acquisition time to measure one AP-MALDI plate was only 18 min, an example of an AP-MALDI-qTOF TIC spectrum of an automated sequence containing 48 measurements is shown in Figure S4.

### 2.6. AP-MALDI-qTOF-MS Settings

Mass spectra were recorded using a Compact qTOF MS (Bruker Daltonics, Bremen, Germany; resolution 25,000 FWHM at *m/z* 1200) equipped with an AP-MALDI (ng) UHR system (MassTech Inc., Columbia, MD, United States) fitted with a Nd:YAG 355 nm laser using a frequency of 10 kHz and 35% laser power. The AP-MALDI software was set to spiral mode, screening an area of approximately 0.6 mm^2^ using an automated sequence. Each well was measured for 15 s. The measurement was performed in both positive and negative ionization modes. Internal and external calibration was performed using a perfluoroalkyl sulfonic acids (PFSA) calibration mixture containing C4, C6, and C8 with a concentration of 1000 mg L^−1^ in acetonitrile over a mass range of *m/z* 299 to 1037 in negative ionization mode, and *m/z* 377 to 1115 in positive ionization mode. The qTOF-MS parameters and more details on the PFSA calibration mix are given in the Supplementary Materials (see Tables S3 and S4 and Figures S5 and S6).

Compass DataAnalysis software (Bruker Daltonics, Bremen, Germany) was used to perform internal calibration of the obtained PFSA mass spectra and TASQ 1.4 software (Bruker Daltonics, Bremen, Germany) was used for the screening of plastic additives. A combined database was created from the EPA Flame-retardant [39] and plastic Norman CPPdb [40] databases containing 1366 unique compounds with 1035 unique molecular formulas. The [M+H]^+^, [M+K]^+^, [M+Na]^+^, and [M+O+K]^+^ adducts, respectively, were added for positive ionization mode, while [M-H]^−^ ions, fragmentation ions obtained from standards solutions (Table S2), and [M+Cl]^−^ adducts for chlorinated paraffins were included for negative ionization mode. Identification of the additives was based on the following criteria: mSigma <100, maximum mass deviation <5 mDa, and peak height >3 times the highest signal obtained for the blanks (*n* = 3; each plate contained 3 blanks). The mSigma value is a measure of the correlation (goodness-of-fit) calculated between the theoretical and measured isotopic pattern (mSigma <100 acceptable, <50 good, and <25 excellent) [15]. Each sample was analyzed in triplicate, and identification was only validated if the plastic additive was detected in all three measurements. Identification was based on confidence level 4 according to the scheme of Schymanski et al. [41], which implies identification on exact mass and isotopic pattern (mSigma).

## 3. Results and Discussion

A special GNP-doped AP-MALDI target plate was designed to aid with the AP-MALDI-qTOF-MS method for the fast-screening of additives in electronics and plastic consumer products. During the design optimization of the GNP-doped AP-MALDI target plate, the following parameters were considered: type of self-curing resin, target well-depth, and the GNP concentration. In this study, we first investigated if a similar 3D-printing procedure, as previously described by Wang et al. [27], could be used to fabricate GNP-doped target plates for AP-MALDI.

Target AP-MALDI plates were 3D-printed with GNP levels ranging from 0.01 wt.% to 0.1 wt.%, as described by Wang et al. [27]. A standard mixture (Table S1) was used to assess the performance of the target plates, the mass spectra are shown in Figure S7. The highest GNP concentration (0.1 wt.%) showed increased signal intensities in both positive and negative ionization modes. However, we observed that a higher GNP content pipetted on a stainless-steel AP-MALDI plate showed better AP-MALDI performance than the best performing GNP-doped 3D-printed target plate. Due to insufficient curing of the 3D-photocuring resin [27], we were not able to reproducibly produce 3D-print target plates with GNP levels above 0.1 wt.%.We subsequently explored an alternative technique to fabricate the GNP-doped AP-MALDI target plates, by using resin-casting as an alternative to 3D-printing (see Supplementary Materials, Figures S1–S3).

### 3.1. Suitable Self-Curing Resin

Three different synthetic resins, epoxy-, PUR-, and UP resin, were evaluated to cast AP-MALDI target plates impregnated with GNP. After curing, the UP plates showed minor shrinkage and were visibly warped. To prevent the UP plates from warping, they were clamped on a flat surface directly after the casting procedure. The PUR and epoxy plates showed no visible shrinkage or warping. After curing, all plates were washed with toluene to remove uncured resin residue. To evaluate the effect of sufficient cleaning of the target plates on the AP-MALDI-TOF signal intensity, a standard mixture (Table S1) was analyzed on a GNP-doped UP target plate before and after cleaning. The results of the effect on signal intensity are shown in Figure S8. The UP and epoxy plates showed good performance, with no signs of degradation after several laser shots. In contrast, the PUR plates showed severe signs of polymer degradation in the form of visible laser marks as well as interference peaks on the mass spectra in the low molecular weight region. Even though epoxy resin has slightly better overall characteristics, UP plates were used for all further experiments because the manufacturing of these plates was significantly quicker (UP plates cured within 10 min compared to 24 h for the epoxy target plates). More detailed information about the production and cleaning of the target plates is given in the Supplementary Information.

### 3.2. Well-Depth of the Target Plate

The AP-MALDI target plate design was further optimized by including dedicated sample wells to minimize sample-to-sample contamination and increase the sample deposition volume to increase the signal intensity of the additives. The depth of the sample wells, which had a significant influence on the measurable and visible surface area in the wells, was limited by the shallow 28° angle of the laser beam and the shallow angle of the built-in AP-MALDI camera relative to the AP-MALDI plate. As shown in Figure S9, a visual representation is given of the sample well on the target plate, showing the decrease in measurable and visual area in the deeper wells. Due to the narrow tolerances, deeper wells can block the laser beam, resulting in the complete loss of the MS-signal. Increasing the diameter of the wells could solve this problem but this would have resulted in fewer wells per plate and lower overall throughput. Therefore, an optimum well-depth of 0.3 mm was selected, which was used for all further experiments. In total, the MALDI target plate contained 48 wells with a width of 3.2 mm, as shown in Figure S3.

### 3.3. GNP Concentration of the AP-MALDI Target Plate

**Figure 1:**
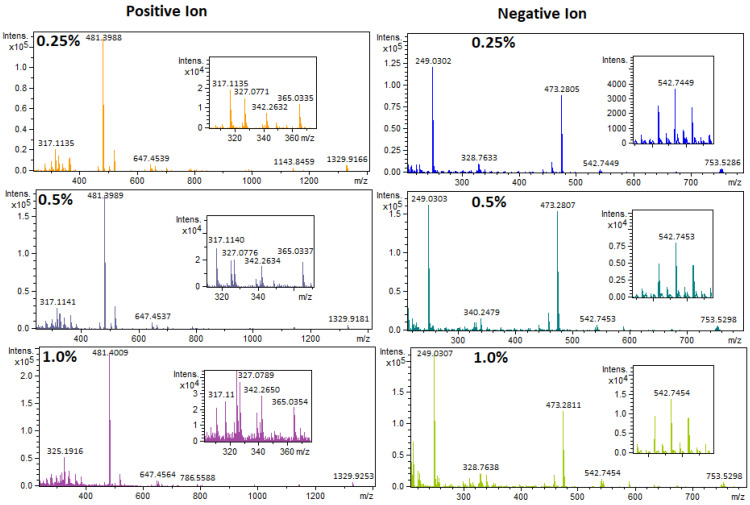
AP-MALDI-qTOF-MS full-scan mass spectra of a standard mixture (Table S1) measured on three different GNP-doped UP AP-MALDI target plates. Top; 0.25 wt.% GNP, middle; 0.5 wt.% GNP, and bottom; 1.0 wt.% GNP. Left: positive ionization mode. Right: negative ionization mode.

Following the plate composition and design, the optimum GNP content of the AP-MALDI target plate needed to be defined. The ionization mechanism of GNPs has not been studied in detail [30,34,42]. However, the unique properties of GNPs have shown to decrease heat capacity, increase surface area and surface UV absorbance, and therefore increase the laser desorption/ionization efficiency [43]. Since a mixture of synthetic resin and GNPs was used, the properties of the target plate could be modified. A higher GNP concentration could improve the trapping of analytes per surface area, decreased heat capacity, and increased surface UV absorbance [43]. However, excessively high GNP concentration could lead to increased heat conductivity and poor physical properties of the plate itself. The latter could lead to warping or cracking of the target plates after or during curing. To optimize the GNP concentration, three UP target plates were fabricated containing 0.25 wt.%, 0.50 wt.%, and 1.0 wt.% GNPs. The mixing of the GNP with the UP resin, and the casting procedure, are described in the Supplementary Materials (also see Figures S1 and S2). In Figure 1, the mass spectra are shown of a standard mixture (Table S1) measured on these three UP target plates. The standard mixture contained 80 mg L^−1^ TBBPA, 110 mg L^−1^ TPhP, 150 mg L^−1^ DBP, 140 mg L^−1^ Irgafos 168, 140 mg L^−1^ Tinuvin 770, and 140 mg L^−1^ TTBP-TAZ in toluene. The highest GNP content (1.0 wt.%) in the plates resulted in the highest analyte signal intensity. This result was similar in both positive and negative ionization modes, which is not in agreement with Wang et al. [27], who observed optimal performance using 0.1 wt.% graphene for the negative ionization mode and 0.02 wt.% graphene for the positive ionization mode [27]. The differences in results could be due to the lower laser frequency used for the desorption and ionization of analytes with the vacuum MALDI system compared to the AP-MALDI system used in this study. The higher GNP content needed for AP-MALDI was also the reason why 3D-printing could not be used to fabricate the GNP-doped target plates. As shown in Figure 1, the highest responses were achieved using an AP-MALDI target plate with a GNP concentration of 1.0 wt.%, but the signal to noise ratio was lower in the 0.5 wt.% GNP AP-MALDI target plate, especially in the lower *m/z* region. Therefore, the AP-MALDI target plate with 0.5 wt.% GNP was selected as the optimum GNP concentration, showing the best overall performances, high AP-MALDI response, low noise levels, and good physical properties.

### 3.4. Analysis of Standards Using the Optimized GNP-Doped UP Target Plates

To assess the performance of the optimized GNP-doped UP AP-MALDI target plates for the screening of plastic additives, thirty different standards of common plastic additives were measured in the concentration range of 100 to 1000 mg L^−1^ (Table S2). The positive adducts [M+H]^+^, [M+Na]^+^, and [M+K]^+^ were observed for most analytes, with [M+Na]^+^ peaks often at a significantly lower intensity than the [M+H]^+^ and [M+K]^+^ adduct signals. For triphenylphosphine and Irgafos 168, the original structure was barely detectable as these compounds could oxidize upon ionization [44,45]. BPA only showed a peak at *m/z* 213.0916 in positive ionization mode, indicating the loss of a methyl group. Tinuvin 770 and Uvitex OB were very efficiently ionized with minimal sodium or potassium adduct formation. For Tinuvin 770, the most abundant peak was [M+H]^+^ and Uvitex OB was only observed as [M]^+^ in positive ionization mode. The observed negative ions (Table S2) include [M-H]^−^, [M+Cl]^−^ for chlorinated analytes and [M+Br]^−^ or [M-Br+O]^−^ for brominated analytes. In negative ionization mode, resorcinol bis (diphenyl) phosphate (RDP), bisphenol-A bis (diphenyl phosphate) (BDP), and TPhP showed a characteristic loss of a benzene group [M-C_6_H_5_]^-^. Irgafos 168 oxidized and lost a di-tert-butylphenyl group [M-C_14_H_21_+O]^−^, where TTBP-TAZ lost a tribromophenol group [M-C_6_H_2_Br_3_-H]^−^ [46,47]. The advantage of AP-MALDI-qTOF-MS is that, in general, less fragmentation was observed; however, the analyte-specific fragmentation can make it challenging to screen analytes based solely on their molecular formula.

### 3.5. Reproducibility

Reproducibility in conventional dried droplet (AP-)MALDI analysis is often a source of concern, due to matrix aggregation or the formation of inhomogeneous matrix crystals. However, using immobilized GNP in the doped MALDI plates, these problems were largely eliminated [27]. To determine the reproducibility of the method, three consumer plastic products (a TV housing unit, a heat sealer, and a Christmas ornament) were extracted in triplicate. These samples have previously been analyzed and contain various organophosphate and BFRs [48]. The samples were extracted in triplicate and each extract was added into three different wells on the GNP-doped AP-MALDI plate and measured in positive and negative ionization modes. A comprehensive overview of the calculated relative standard deviations (RSD) is given in Table 1. For the calculation of the extract to extract RSDs, the average peak heights of the triplicate analysis were used (*n* = 3). To calculate the spot-to-spot RSDs (*n* = 9), the average spot to spot RSDs within each extract (*n* = 3) were used. In total, nine plastic additives were detected in the TV housing unit, whereas the heat sealer and the Christmas ornament contained 10 and 15 additives, respectively. The extract-to-extract RSDs of the triplicate analysis of the plastic additives in the TV housing unit, heat sealer, and Christmas ornament ranged from two to 33%, one to 28%, and three to 46%, respectively. The relatively higher extract-to-extract RSDs observed in the Christmas ornament may be related to the inhomogeneity of the sample. The additives in the TV housing unit and the heat sealer, generally high-quality plastics, are added to the products for a purpose (e.g., flame retardancy) and are therefore homogeneously distributed through the samples, whereas in the Christmas ornament, fabricated from low-quality plastic, the additives are possibly coming from a WEEE fraction that has been recycled into the products and are therefore less homogeneously distributed through the sample. Only for BDP, RDP, and DBP measured in the positive ionization mode, the extract to extract RSDs for the [M+K]^+^ ion were relatively high: 46%, 44%, and 43%, respectively. However, for BDP and BDP measured in the negative ionization mode, the extract-to-extract RSDs of the [M-C_6_H_5_]^-^ ions were 24 and 33%. The spot-to-spot RSDs of the plastic additives in the TV housing units, heat sealer, and Christmas ornament ranged from 10 to 30%, 6 to 48%, and 11 to 31%, respectively. High spot-to-spot RSD (48%) was observed for TBBPA in the heat sealer, which may have been caused by the relatively high TBBPA concentration (70.000 mg L^−1^, 7 wt.%) in this sample. The high TBBPA concentration may not be efficiently extracted from the sample. This also explains why the extract-to-extract RSD is acceptable (1%) and why only the spot-to-spot RSD is high (48%). Overall, the acceptable RSDs are in the same range as observed by Wang et al. [27] - (21 to 39% for spot to spot (*n* = 20), 20 to 42% for extract to extract (*n* = 15)), measuring standard solutions on the 3D-printed graphene-doped target using a vacuum MALDI system.

The between plate reproducibility was calculated using the PFSA mixture used to calibrate the qTOF system. While measuring plastic samples and standards, a PFSA mixture (Table S4) was measured at the beginning and end of each automated sequence. Four plates were used to perform the measurements shown in this study. The measurements were performed over two different days. The average reproducibility between the plates (*n* = 4) was 41% and 36% in negative and positive ionization modes, respectively (Table S6). To further increase extract-to-extract and spot-to-spot RSDs and the between plate reproducibility, a labeled injection/internal standard could be added to each extract to correct for the variations of the AP-MALDI-TOF analysis.

### 3.6. Analyses of Plastic Samples with Known Concentrations Using the Optimized UP GNP-Doped Targets

To evaluate the performance of the AP-MALDI-qTOF-MS screenings method, six plastic consumer products were screened which have been previously quantified for a variety of flame retardants [48,49]. These samples include three TV housing units, a heat sealer housing, a (non-electric) Christmas ornament, and a travel power adapter. Additionally, a PVC-certified reference material (CRM) has been included (KRISS 113-03-006) which contains 0.1 wt.% DBP, 0.1 wt.% BBP, 0.1 wt.% DEHP, 0.1 wt.% DNOP, 0.1 wt.% dimethyl phthalate (DMP), and 0.1 wt.% diethyl phthalate (DEP). Triplicate extraction was performed for samples two, three, and four, and all other samples were extracted once. The screening results of the six plastic consumer products measured in triplicate with the AP-MALDI-qTOF-MS are shown in Table 2.

**Figure 2:**
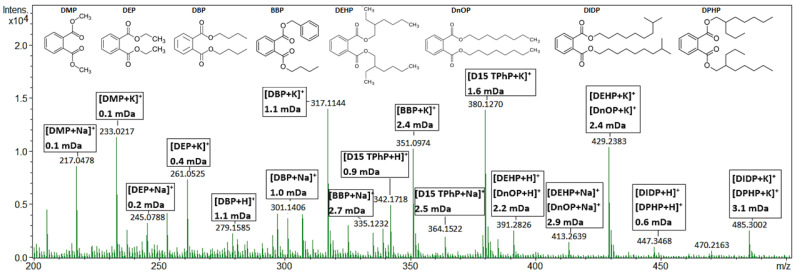
AP-MALDI-qTOF-MS full scan mass spectrum of a PVC CRM (KRISS 113-03-006) extract, measured on a GNP-doped AP-MALDI target. The extract has been spiked with 125 mg L^−1^ labeled D15-TPhP. Peaks are annotated with their molecular ion and mass error in mDa. All annotated peaks have mSigma values below 100.

All additives previously quantified [48,49] in the plastic consumer products using GC-MS and LC-MS/MS were confirmed with the AP-MALDI using the optimized UP GNP-doped target plate, except for TCP in the Christmas ornament and BDE209 (Table 1). The TCP level in the Christmas ornament was 50 mg L^−1^, which is too low for the detection with the AP-MALDI-qTOF-MS method. Further research is needed to investigate why BDE209 could not be detected using AP-MALDI-qTOF-MS. No false-positive detections were found. The additives present in the sub-ppm range could be easily detected with the AP-MALDI. For example, TPhP was detected at 40 mg L^−1^ (0.004 wt.%) in sample two, in both positive and negative ionization mode, tris(2-chloroethyl) phosphate TCEP with a concentration of 1000 mg L^−1^ (0.1 wt.%) in sample six, and 2,4,6-TBP and TBBPA were detected in samples two, four, and six at levels of 3000 mg L^−1^ (0.3 wt.%). BDP and TBPP-TAZ quantified in samples two and 12 were also confirmed with AP-MALDI. Samples thee, four, six, and eight were not previously quantified for RDP and BDP; however, with the AP-MALDI, RDP was detected in all of these samples and BDP in three of them. A positive full scan mass spectrum of the PVC-CRM is shown in Figure 2 and concentrations of 1000 mg L^−1^ (0.1 wt.%) for DBP, BBP, and DEP could easily be detected. DEHP and DNOP were also detectable in the PVC CRM, but we were unable to separate them based on a similar molecular formula. DMP did not meet the set screening criteria and was present as an impurity in the UP GNP-doped target plate. Labeled D15-TPhP (125 mg L^−1^) was added to the extract as a control standard.

### 3.7. Application to Consumer Products

After optimization, the AP-MALDI-qTOF-MS method was used to screen 18 consumer products (Table S7). The high-resolution MS data were screened against the combined database, as previously discussed.

**Figure 3:**
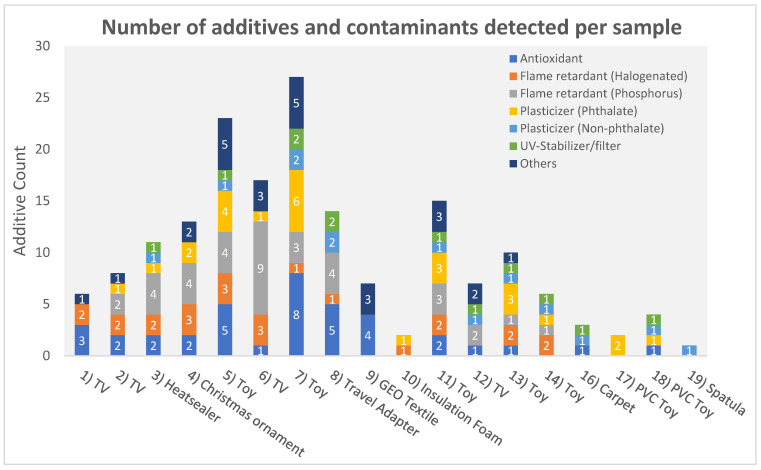
Number and type of additives and contaminants identified per sample. Sample 15 (PVC CRM) is not included in this figure because it is not a consumer product.

In total, 56 additives and other compounds were identified in the samples, including antioxidants (*n* = 14), BFRs (*n* = 5), phosphorus flame retardants (OPFRs) and plasticizers (*n* = 11), phthalate plasticizers (*n* = 7), non-phthalates plasticizers (*n* = 7), UV-stabilizers/UV-filters (*n* = 5), and others (7) (Figure 3). A detailed list of all detected compounds is shown in the Tables S8 and S9.

**Figure 4:**
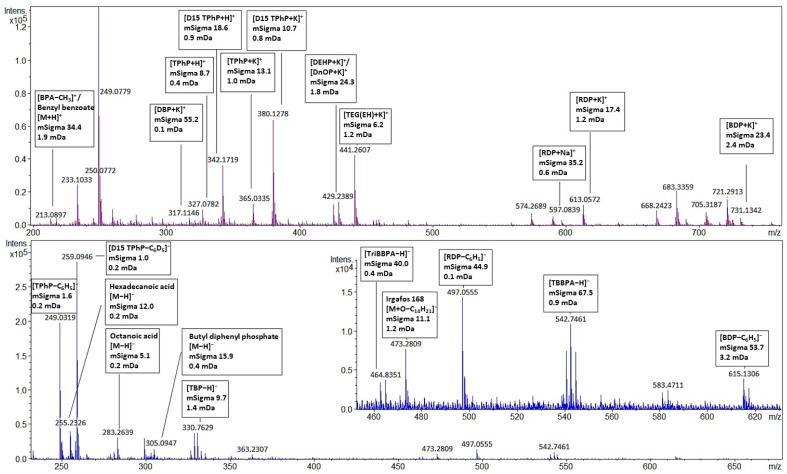
AP-MALDI-qTOF-MS full scan spectrum of sample five with a selected set of annotated compounds. A full set of annotated compounds can be found in Tables S8 and S9) Upper panel: positive ionization mode; lower panel: negative ionization mode. Molecular ions are annotated with their mSigma and mass accuracy (mass error) in mDa. All annotated peaks meet the screening requirements.

The screening results of two children’s toys, a puzzle cube (no. 5), and a toy car (no. 7), showed the presence of over 20 additives. Multiple regulated phthalates (DINP, DPENP, DIBP, DEHP, BBP, DIDP, and DBP) were identified in different children’s toy samples (more details can be found in Tables S8 and S9). The identification of OPFRs and BFRs in plastic children’s toys (no. 5, 7, 11, 13 and 14) may indicate that these products could contain a recycled fraction of WEEE [50]. This improper recycling may lead to unnecessary exposure of children to FRs and other additives [51,52,53]. Potentially toxic additives present in these children’s toys may exceed EU and US regulatory levels [10,12]. However, further quantification of the levels is needed to confirm this. The positive and negative mode full scan mass spectra of the puzzle cube (no. 5) are shown in Figure 4 for a selected set of annotated compounds. A full list of all the annotated compounds can be found in Tables S8 and S9. As a control, each sample was spiked with 125 mg L^−1^ labeled D15-TPhP. The identified peaks are annotated with their molecular ion, their mSigma value, and the mass error in mDa. Although more than 20 compounds were identified in the puzzle cube (no. 5), there are still unidentified peaks present. This indicates that expanding the screening database may further increase the screening capability of the method.

All samples that screened positive for RDP or BDP also contained TPhP, which is in accordance with the literature [54]. In negative ionization mode, Irgafos-168, TPhP, RDP, and BDP are more sensitive but less selective because only fragments are detected. The mass fragment of TPhP [M-C_6_H_5_]^-^ (*m/z* 249.0317) could also be derived from compounds with a similar structure such as diphenyl phosphate [M-H]^-^ or meta-hydroxy-triphenyl phosphate [M-C_6_H_4_OH]^-^. Screening with the AP-MALDI-qTOF-MS was performed solely on specific mass and isotope ratio; no distinction could be made between isomers. For example, BPA showed a signal at [M-CH_3_]^+^ *m*/*z* 213.0916 which has the same molecular formula as benzyl benzoate [M+H]^+^ ion. The flame retardant allyl 2,4,6-tribromophenyl ether (ATE) shows a peak at [M-C_3_H_5_]^-^ *m*/*z* 328.7635 which also corresponds with 2,4,6-TBP [M-H]^-^. In addition, some phthalates with the same molecular formula could not be resolved. Further research could investigate AP-MALDI-qTOF-MS with MS/MS fragmentation using collision-induced dissociation (CID) in combination with sophisticated non-target screening software to increase the screening capabilities of the method. Highly brominated compounds could not be recognized by the screening software. According to Ballesteros et al. [15], compounds such as TTBP-TAZ cannot be recognized by TASQ due to isotopic pattern fit limitations in the software. A similar problem was encountered in samples three and eight, which contained high concentrations (>10,000 mg L^−1^) of TBBP-A. These compounds have therefore been manually identified.

## 4. Conclusions

An innovative GNP-doped UP target plate was fabricated and optimized for the AP-MALDI system to simplify sample preparation and increase signal intensity and reproducibility. The target plates can be fabricated with both UP and epoxy resin, which indicates that UV-stable thermoplastic polymers could be used to produce relatively cheap, high volume, injection-molded GNP-doped (AP-) MALDI target plates. The target plate was used to develop a novel fast-screening method to confirm the presence of additives in plastic consumer products using AP-MALDI-qTOF-MS. The analysis time was 15 s per measurement using an automated sequence.

The results showed that AP-MALDI-qTOF-MS could detect additives in plastic consumer products in the sub-ppm range. This indicates that the screening method can be used as an initial screening to verify the compliance to plastic additive (e.g., phthalates and flame retardants) regulations. This is the first study where AP-MALDI was used in a fast-screening application for additives in plastic products.

## Figures and Tables

**Table 1:** Spot-to-spot and extract-to-extract RSD(%) of three plastic samples. The RDP and TriBBPA levels in sample three and the TEG-EH levels in sample four were relatively low; therefore, in some of the measurements, the quantifier ion was not visible, resulting in a non-detect. (*) spot-to-spot RSD(%) based on n=8 instead of n=9. (**) spot-to-spot RSD(%) based on n=7 instead n=9. (-) Analyte not detected in sample). (^$^) analytes have not been verified with standards.

		2 (TV Housing Unit)		3 (Heat Sealer)		4 (Christmas Ornament)	
Analyte	Adduct Ion	Spot-to-Spot RSD (%) (*n* = 9)	extract-to-extract RSD (%) (*n* = 3)	spot-to-spot RSD (%) (*n* = 9)	extract-to-extract RSD (%) (*n* = 3)	spot-to_spot RSD (%) (*n* = 9)	extract-to-extract RSD (%) (*n* = 3)
TPhP	[M+K]^+^	16	5	12	19	11	12
	[M+H]^+^	12	2	26	28	12	3
	[M-C_6_H_5_]^−^	14	13	14	4	18	19
BDP	[M+K]^+^	20	6	15	23	18	46
	[M-C_6_H_5_]^−^	16	10	18	5	22	24
RDP	[M+K]^+^	-	-	* 6	10	19	44
	[M-C_6_H_5_]^−^	-	-	13	3	25	33
TBBPA	[M-H]^−^	-	-	48	1	15	18
TTBP-TAZ	[M-C_6_H_2_Br_3_-H]^−^	10	13	-	-	-	-
2,4,6-TBP	[M-H]^−^	17	13	-	-	31	5
Irgafos 168	[M-C_14_H_21_+O]^−^	16	3	23	14	24	19
Sulisobenzone ^$^	[M-H]^−^	-	-	21	19	-	-
BDPHP ^$^	[M-H]^−^	-	-	14	2	13	25
BHT-COOH ^$^	[M-H]^−^	-	-	26	24	-	-
Irganox 1076 ^$^	[M+K]^+^	15	15	-	-	16	11
ATBC ^$^	[M+K]^+^	-	-	-	-	21	17
DEHP ^$^	[M+K]^+^	12	10	10	17	11	13
DBP ^$^	[M+K]^+^	-	-	-	-	11	43
TEG-EH ^$^	[M+K]^+^	19	10	-	-	** 18	15
TriBBPA ^$^	[M-H]^−^	-	-	** 33	17	28	26
Palmitic acid ^$^	[M-H]^−^	30	33	-	-	21	3
Stearic acid ^$^	[M-H]^−^	-	-	-	-	19	8

**Table 2:** The results of the seven plastic products analyzed with the AP-MALDI-qTOF-MS. Shown in times detected per times measured (n/3 or n/9). The OPFR and BFR concentrations given in wt.% show in this table are from previous work [48,49]. - = Not analyzed.

Analyte		BDP	RDP	TPhP	TCEP	TCP	TTBP-TAZ	2,4,6-TBP	TBBPA	BDE209
CAS-No.		5945-33-5	57583-54-7	115-86-6	115-96-8	1330-78-5	25713-60-4	118-79-6	79-94-7	1163-19-5
Adduct Ion		[M+K]^+^	[M+K]^+^	[M+K]^+^	[M+K]^+^	[M+K]^+^	[M - C_6_H_2_Br_3_-H]^−^	[M-H]^−^	[M-H]^−^	
**2 (TV-housing)**	GC/LC-MS/MS (% wt.)	0.5	<0.002	0.004	-	-	12	0.3	<0.005	0.1
	AP-MALDI (n/n)	9/9	0/9	9/9	0/9	0/9	9/9	9/9	0/9	0/9
**3 (Heat sealer)**	GC/LC-MS/MS (% wt.)	-	-	0.03	<0.002	<0.002	-	-	7.0	0.5
	AP-MALDI (n/n)	9/9	8/9	9/9	0/9	0/9	0/9	9/9	9/9	0/9
**4 (Christmas ornament)**	GC/LC-MS/MS (% wt.)	-	-	0.09	<0.002	0.005	-	-	0.3	0.9
	AP-MALDI (n/n)	9/9	9/9	9/9	0/9	0/9	0/9	9/9	9/9	0/9
**6 (TV-housing)**	GC/LC-MS/MS (% wt.)	-	-	0.4	0.1	<0.002	-	-	0.3	8.9
	AP-MALDI (n/n)	3/3	3/3	3/3	3/3	0/3	0/3	0/3	3/3	0/3
**8 (Travel adapter)**	GC/LC-MS/MS (% wt.)	-	-	0.04	<0.002	<0.002	-	-	6.9	1.8
	AP-MALDI (n/n)	0/3	3/3	3/3	0/3	0/3	0/3	0/3	3/3	0/3
**12 (TV-housing)**	GC/LC-MS/MS (% wt.)	13	<0.02	0.2	-	-	<0.01	<0.01	<0.05	<0.02
	AP-MALDI (n/n)	3/3	0/3	3/3	0/3	0/3	0/3	0/3	0/3	0/3

## Data Availability

The data presented in this study are available on request from the corresponding author.

## Supplementary Materials

The following Supplementary Materials can be downloaded at: https://www.mdpi.com/article/10.3390/toxics11020108/s1, Figure S1: fabrication of the GNP doped AP-MALDI target plates; Figure S2: photographs of the silicone mould; Figure S3: Casted GNP doped UP AP-MALDI target plate; Figure S4: Total ion current plot of an automated sequence containing 48 measurements; Figure S5: PFSA-mixture analysed on a GNP-doped UP AP-MALDI target plate (0.5 wt.% GNP) in negative ionisation mode; Figure S6: PFSA-mixture analysed on a GNP- doped UP AP-MALDI target plate (0.5 wt.% GNP) in positive ionisation mode; Figure S7: Left: 0.01 wt.% GNP 3D-printed target. Middle: 0.1 wt.% GNP 3D-printed target. Right: metal plate with a layer of GNP; Figure S8: Performance of 0.5 wt.% GNP-doped UP AP-MALDI target plate before and after cleaning; Figure S9: Schematic illustration of, the influence of well-depth on AP-MALDI analysis; Figure S10: Results of using different solvent(systems) for the extraction of 3 plastic samples after 10 min of sonicating; Figure S11: Effect of extracting three plastic samples for 1 night using different solvent systems on the AP-MALDI-TOF performance; Figure S12: Effect of adding solvents separately on AP-MALDI-TOF performance; Table S1: Standard solution mixture to optimize the GNP doped AP-MALDI plates; Table S2: Standards with their most abundant ions, measured on a GNP doped UP target plate using AP-MALDI-TOF; Table S3: qTOF MS Parameters; Table S4: Ions used to calibrate the qTOF systems; Table S5: Between plate reproducibility by measuring the PFSA mixture; Table S6: Description of the screened plastic products; Table S7: Tentatively identified additives in the screened products using AP-MALDI screening negative ionisation mode; Table S8: Tentatively identified additives in the screened products using AP-MALDI in positive ionisation mode.
