# Supplementary material for: A Rapid Screening Method for the Detection of Additives in Electronics and Plastic Consumer Products Using AP-MALDI-qTOF-MS
Source: Toxics. 2023 Jan 23;11(2):108. doi: 10.3390/toxics11020108 (PMC9960555; doi:10.3390/toxics11020108)
Supplement: Supplementary file 1 [file toxics-11-00108-s001.zip › toxics-2107605-supplementary.pdf]

Supplementary Material

A rapid screening method for the detection of additives in electronics and plastic consumer products using AP-MALDI-qTOF-MS

Maurice de Jonker, Pim. E. G. Leonards, Marja. H. Lamoree, and Sicco. H. Brandsma.

Amsterdam Institute for Life and Environment (A-LIFE), Section Environment and Health, Vrije Universiteit, Amsterdam, De Boelelaan 1085, 1081HV, Amsterdam, the Netherlands,

*Corresponding author

sicco.brandsma@vu.nl

Table of contents

3D-printing of the GNP-doped AP-MALDI target plates………………………………………………………………S2

Resin Casting of the GNP-doped AP-MALDI target plates……………………………………………………………..S2

Fabrication of the silicone mould……………………………………………………………………………………………….S2

Casting the epoxy and polyurethane resin target plates……………………………………………………………….S3

Casting the unsaturated polyester resin target plates…………………………………………………………………..S4

Standard solution mixture to optimize the GNP-doped AP-MALDI plates…………………………………..S5

Analysis of standards using the GNP-doped Targets……………………………………………………………………..S6

qTOF MS Parameters………...S8

AP-MALDI-TOF Calibration……..S9

Total ion current plot of an automated sequence containing 48 measurements………………………..S10

PFSA-mixture analyzed on a GNP-doped UP AP-MALDI target plate (positive and negative)……….S11

Results 3D-printed GNP-doped AP-MALDI target plates……………………………………………………………..S12

Optimizing the GNP-doped AP-MALDI targets: Cleaning the target plates…………………………………S13

Schematic illustration, the influence of well-depth on AP-MALDI analysis…………………………………S14

Optimization of the extraction method ……………………………………………………………………………………S15

Between plate reproducibility……………………………………………………………………………………………………S17

Description of the screened plastic products……………………………………………………………………………S18

Results AP-MALDI screening negative ionization mode…………………………………………………………………S19

Screening results in positive ionization mode…………………………………………………………………………..S20

3D-printing of the GNP-doped AP-MALDI target plates.

A commercially available desktop stereolithography 3D-printer from Form labs (form 2) was used to print the MALDI plates with a 50 µm layer height from Form labs Photopolymer Clear Resin (FLGPCL04; Somerville, MA, USA). Graphene nanoplatelets (surface area 750 m2 g^{-1}, particle size 2 µm, relative gravity: 2.0 - 2.25 g $(cm^3)^{-1}$ and a thickness of a few nm) were purchased from Sigma Aldrich. A 3D-model of the target plate was designed in Fusion 360 (Autodesk Inc. California, US) based on the dimensions of an OPTI-TOF, 192-well plate. 200 ml of 0.01, 0.1 and 0.5 wt.% GNP in photopolymer resin was prepared by adding the GNP to a glass bottle containing the photopolymer, the mixture was vigorously stirred. Before printing, the resin was sonicated for 10 min. After 3D-printing, the models were rinsed with 2-propanol and cured using UV light and 60°C for 30 min (Form Cure). Hereafter the plates were cut from the print supports and the back was sanded flat. A magnet was used to hold the plate in the AP-MALDI target holder.

Resin Casting of the GNP-doped AP-MALDI target plates.

Three different types of commercially available resins were obtained. Two-part epoxy-based resin, Resion EP101 and EP121 were purchase from polyestershoppen.nl. Part A consists of >70 wt.% Bisphenol A epichlorohydrin, >10 wt.% <30 wt.% Oxirane mono[(C12-14-alkyloxy)methyl] derivatives, and part B consisting of 50-70 wt.% 3-aminomethyl-3,5,5- trimethyl cyclohexylamine, 30-50 wt.% benzyl alcohol. The unsaturated polyester (UP) resin from Albastine containing 25-50 wt.% styrene and Albastine curing paste containing 50 wt.% dibenzoyl peroxide were purchased from the Praxis. Polyurethane (PUR) resin (Axson F160) was purchased from siliconesandmore.com. Two-part silicone rubber (1:1) shore 15, Resion SR1-15A and SR1-15B were purchased from polyestershoppen.nl.

Fabrication of the silicone mould.

To make a silicone mould, a model was required to imprint in the silicone. 3D-printing is an ideal technique to rapidly produce such a model. Because thin SLA 3D-printed models tend to warp after curing an FDM 3D-printer was used. The FDM 3D-printer used was an Ultimaker 2 extended (Ultimaker BV. Geldermalsen, NL) using PLA filament. A model of the AP-MALDI target plate was designed in Fusion 360 (Autodesk Inc. California, US) to fit the AP-MALDI target holder. The front and back of the plate were printed separately. A flat and smooth plastic plate, the thickness of a credit card (0.76mm), was carefully glued in between and cut to shape. This was done to ensure a perfectly flat target plate of which the bottom of each well is flat and smooth. As shown in figure S1 the model was propped up in a plastic cup.

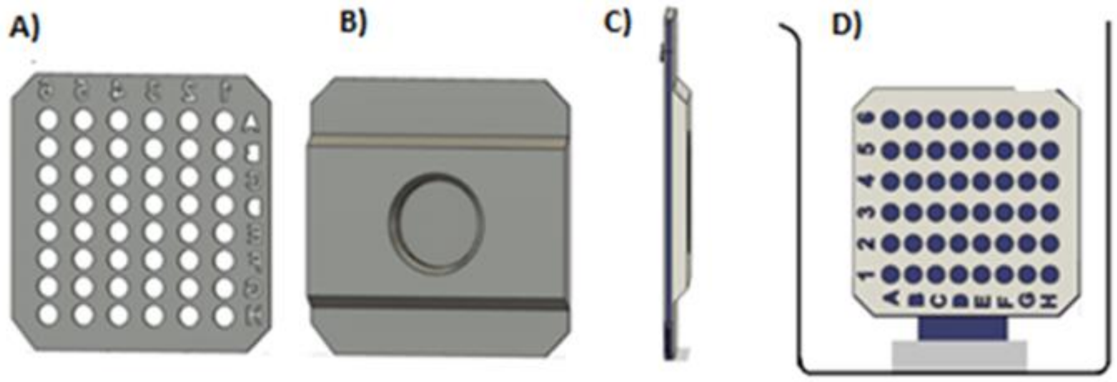

Figure S1: A And B: front and back of the FDM 3D-printed parts. C) Side view of finished model with the smooth plastic card in-between the FDM 3D-printed parts. D).

Two-part silicone resin was mixed as prescribed by the manufacturer and carefully poured around the model. After the silicone was cured the mould was cut open in a zig-zag pattern to aid in realignment when the mould is closed. The finished mould is shown in Figure S2.

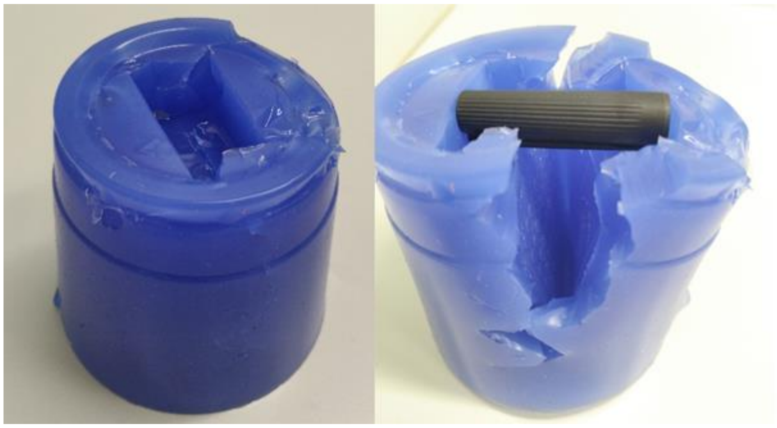

Figure S2: photographs of the silicone mould.

Casting the epoxy and polyurethane resin target plates.

Parts A and B of the two-part resins were separately weighed, in the ratio prescribed by the manufacturer (10A: 6B for the epoxy resin, 1: 1 for the PUR resin). GNPs were added to both parts so that the final product contained 0.5 wt.% GNP and stirred thoroughly. Hereafter the epoxy part A and B were combined and mixt in a third container by manual stirring for 10 minutes. Air bubbles were removed by sonication for 20 min, air bubbles left on the surface were removed using a blow torch. Hereafter, the mixture was poured into the silicone moulds. A spatula was used to agitate the resin inside the moulds, removing air pockets. The epoxy resin casts were left to cure overnight in an oven at 50 °C before unmoulding. The plates were left to cure for two more days at 50 °C. The PUR plates could be removed from the mould after 10 min and were left to cure overnight.

Casting the unsaturated polyester resin target plates.

In earlier experiments aggregation of GNP could be seen in the UP plates, causing unstable MS signals and decreased reproducibility. Therefore, in the casting process of the GNP doped UP plates, the GNPs are pre-dispersed in acetone. This made the plates visibly darker, and no GNP aggregation could be observed. Acetone in these proportions does not seem to have a significant effect on the curing of the UP resin.

To fabricate five GNP doped UP plates, containing 0.5 wt.% GNP, 0.25 g GNP was dispersed in 4 ml acetone by sonicating for 1 hour. The GNP dispersion was added to 50 g UP resin and manually stirred. A light vacuum was used to remove air bubbles. Before casting the resin was divided in 10 g aliquots. Per aliquot 1 g (10 wt.%) of curing paste was stirred in for one minute after which the resin was poured into the silicone mould. A spatula was used to agitate the resin inside the mould, removing air pockets. After 10 min the cured resin plate was removed and allowed to further cure and fume of in a fume hood overnight. A picture of a GNP-doped UP AP-MALDI target plate is shown in Figure S3.

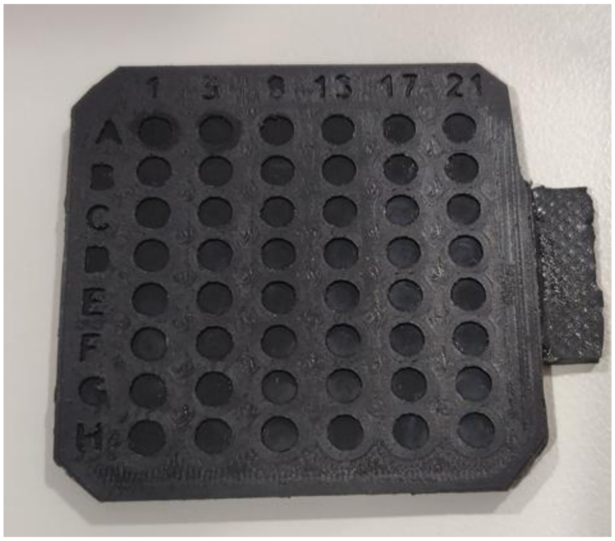

Figure S3: Casted GNP doped UP AP-MALDI target plate containing 48 wells with a width of 3.2 mm and a well depth of 0.3 mm .

Table S1: Standard solution mixture to optimize the GNP-doped AP-MALDI plates.

Concentration (mg L^{-1})	Compound	Chemical formula	Molecular Ion	m/z Theoretical
80	Tetrabromobisphenol A (TBBPA)	$C_{15}H_{12}Br_4O_2$	$[M-H]^-$	542.7451
110	Triphenyl phosphate (TPhP)	$C_{18}H_{15}O_4P$	$[M+K]^+$	365.0345
			$[M+H]^+$	327.0786
			$[M-C_6H_5]^-$	249.0317
150	Dibutyl phthalate (DBP)	$C_{16}H_{22}O_4$	$[M+K]^+$	317.1155
140	Tris(2,4-di-tert-butylphenyl) phosphite (Irgafos 168)	$C_{42}H_{63}O_3P$	$[M+H]^+$	647.4593
			$[M+O+K]^+$	701.4101
			$[M_2+K]+$	1330.8661
			$[M+O–C_{14}H_{21}]^-$	473.2821
140	bis(2,2,6,6,-tetramethyl-4-piperidyl)subacetate (Tinuvin 770)	$C_{28}H_{52}O_4N_2$	$[M+H]^+$	481.4006
140	2,4,6-tris(2,4,6-tribromophenoxy)-1,3,5-triazine (TTBP-TAZ)	$C_{21}H_6Br_9N_3O_3$	$[M-C_6H_2Br_3– H]^-$	753.5292

Analysis of standards using the GNP-Doped Targets.

To assess the applicability of the GNP-doped AP-MALDI targets for the screening of polymer additives and gain understanding about the ionization mechanisms. A variety of standards are prepared in concentrations of 1000 mg L^{-1} and 100 mg L^{-1}. TCEP, TCIP, TDCIPP and F3014 standards are prepared in acetone. PFOS and PFOA standards are prepared in ACN. The other standards (Table S2) are prepared in toluene. 2 µL standard solution was pipetted on a GNP doped UP target plate, air dried and analysed at 10 kHz and 35 % laser power. The highest detected ions are shown in Table S2. BDE209 could not be detected, even at 1000 mg L^{-1}. Irganox 1010, BTBPE, TBBPA-DBDPE and F3014 could be detected at 1000 mg L^{-1} but not at 100 mg L^{-1}. The other listed analytes could be detected at 100 mg L^{-1} (S/N > 3). Identification of the standards was based on the following criteria mSigma <100, maximum mass deviation <5 mDa.

Table S2: Standards with their most abundant ions, measured on a GNP-doped UP target plate using AP-MALDI-TOF.

Additive	Abr.	Cas #	Formula	Major peaks (+)	Major peaks -
**3,3'5,5'-Tetrabromobisphenol A**	TBBPA	79-94-7	$C_{15}H_{12}Br_4O_2$	-	$[M-H]^-$ $[M-H_2Br+O]^-$
**Decabromodiphenyl ether**	BDE209	1163-19-5	$C_{12}Br_{10}O$	-	-
**Hexabromocyclododecane**	HBCD	25637-99-4	$C_{12}H_{18}Br_6$	-	$[M+Br]^-$
**2,4,6-tris(2,4,6-tribromophenoxy)-1,3,5-triazine**	TTBP-TAZ	25713-60-4	$C_{21}H_6Br_9N_3O_3$	$[M+H]^+$, $[M-Br]^+$	$[M - C_6H_2Br_3-H]^-$
**tris(phenyl) phosphate**	TPhP	115-86-6	$C_{18}H_{15}O_4P$	$[M+H]^+$, $[M+K]^+$, $[M+Na]^+$	$[M-C_6H_5]^-$
**Benzyl butyl phthalate**	BBP	85-68-7	$C_{19}H_{20}O_4$	$[M+H]^+$, $[M+K]^+$, $[M+Na]^+$	-
**Dibutyl phthalate**	DBP	84-74-2	$C_{16}H_{22}O_4$	$[M+H]^+$, $[M+K]^+$, $[M+Na]^+$	-
**1H,1H,2H,2H-Perfluorooctanesulfonic acid**	PFOS 6:2fts	27619-97-2	$C_8H_4F_{13}O_3SNa$	$[M+Na]^+$, $[M_2+Na]^+$	$[M-Na]^-$, $[M_2-Na]^-$
**Perfluorooctanesulfonic acid**	PFOS	45298-90-6	$C_8F_{17}SO_3K$	$[M+K]^+$, $[M_2+Na]^+$	$[M-K]^-$, $[M_2-K]^-$
**Perfluorooctanoic Acid**	PFOA	335-67-1	$C_8HF_{15}O_2$	-	$[M-H]^-$, $[M-COOH]^-$, $[M_2-H]^-$
**Tris(2,4-di-tert-butylphenyl)phosphite**	Irgafos 168	31570-04-4	$[(C_4H_9)_2C_6H_3O]3P$	$[M+O+K]^+$, $[M+Na+O]^+$, $M+H]^+$, $[M+O+H]^+$	$[M+O–C_{14}H_{21}]^-$
**Pentaerythritol tetrakis(3,5-di-tert-butyl-4-hydroxyhydrocinnamate)**	Irganox 1010	6683-19-8	$C_{73}H_{108}O_{12}$	$[M+K]^+$, $[M+Na]^+$	$[M-H]^-$
**Bis(2,2,6,6-tetramethyl-4-piperidyl) sebacate**	Tinuvin 770	52829-07-9	$C_{28}H_{52}N_2O_4$	$[M+H]^+$, $[M+K]^+$, $[M+Na]^+$	-
**2,5-Bis(5-tert-butyl-benzoxazol-2-yl)thiophene**	Uvitex OB	7128-64-5	$C_{26}H_{26}N_2O_2S$	$[M]^+$	$[M-H]^-$
**Bisphenol A**	BPA	80-05-7	$C_{15}H_{16}O_2$	$[M-CH_3]^+$	-
**4-Nonylphenol**	NP	84852-15-3	$C_{15}H_{24}O$	-	$[M-H]^-$
**Triphenyl phosphine**	PPH_3	603-35-0	$C_{18}H_{15}P$	$[M+O+K]^+$	-
**triphenyl phosphine oxide**	PPH_3O	791-28-6	$C_{18}H_{15}OP$	$[M+K]^+$	-
**Tris(2-chloroethyl) phosphate)**	TCEP	115-96-8	$C_6H_{12}Cl_3O_4P$	$[M+H]^+$, $[M+K]^+$, $[M+Na]^+$	-
**tris(2-chloor-1-methylethyl)phosphate**	TCIPP	13674-84-5	$C_9H_{18}Cl_3O_4P$	$[M+H]^+$, $[M+K]^+$, $[M+Na]^+$	-
**Tris(1,3-dichloro-2-propyl)phosphate**	TDCIPP	13674-87-8	$C_9H_{15}Cl_6O_4P$	$[M+H]^+$, $[M+K]^+$, $[M+Na]^+$	$[M+Cl]^-$, $[M-C_3H_5Cl_2]^-$

**Bisphenol-A bis(diphenyl phosphate)**	BDP	5945-33-5	$C_{39}H_{34}O_8P_2$	$[M+H]^+$, $[M+K]^+$, $[M+Na]^+$	$[M-C_6H_5]^-$
**Resorcinol bis(diphenyl phosphate)**	RDP	57583-54-7	$C_{30}H_{24}O_8P_2$	$[M+H]^+$, $[M+K]^+$, $[M+Na]^+$	$[M-C_6H_5]^-$
**Bisphenol A diglycidyl ether**	BPA diglicyl ether	1675-54-3	$C_{21}H_{24}O_4$	$[M+K]^+$, $[M+Na]^+$	-
**2,4,6-Tribromophenol**	2,4,6-TBP	118-79-6	$C_6H_3Br_3O$	-	$[M-H]^-$
**Allyl 2,4,6-tribromophenyl ether**	ATE	3278-89-5	$C_9H_7Br_3O$	-	$[M-C_3H_5]^-$
**Hexabromobenzene**	HBB	87-82-1	C_6Br_6	-	$[M-Br+O]^-$, $[M-Br_2+OH]^-$
**3,3'-((propane-2,2-diylbis(2,6-dibromo-4,1-phenylene))bis(oxy))bis(1-(2,4,6-tribromophenoxy)propan-2-ol)**	F3014, TBP-CMOTBBPA-CMO-TBP	158725-44-1	$C_{33}H_{26}Br_{10}O_6$	-	$[M+Br]^-$
**Tetrabromobisphenol A bis(dibromopropyl ether)**	TBBPA-DBDPE	21850-44-2	$C_{21}H_{20}Br_8O_2$	-	$[M+Br]^-$, $[M-Br_2]^-$
**1,2-Bis(2,4,6-tribromophenoxy)ethane**	BTBPE	37853-59-1	$C_{14}H_8Br_6O_2$	$[M+Na]^+$, $[M+K]^+$	-

Table S3: qTOF MS Parameters.

	**Negative**	**Positive**	**Funnel 1 RF**	150 Vpp	**Funnel 2 RF**	200 Vpp
**Source**	Nano ESI online		**isCID**	0 eV	**Hexapole RF**	50 Vpp
**End plate offset**	200 V		**Ion Energy**	4 eV	**Low Mass**	100 m/z
**Capillary**	1500 V	2000 V	**Collision Energy**	7 eV	**Collision RF**	850 Vpp
**Dry gas N_2**	2 l/min		**Transfer Time**	80 us	**Pre-Pulse Storage**	5 us
**Dry gas Temp**	220 °C		**mass range:** 100 - 1600 m/z		**Spectra rate:** 0.4 Hz	

AP-MALDI-qTOF Calibration.

To obtain reliable data with low mass errors it is important to calibrate the qTOF-MS frequently. The most reliable method of obtaining data with low mass errors is to measure an internal calibrant in every spectrum. It was noticed that perfluoroalkyl sulfonic acids (PFSAs) ionize exceptionally well on the AP-MALDI using GNP matrix, especially in negative ion mode. Therefore, a mixture containing 1000 mg L^{-1} C4, C6, and C8 PFSA in ACN was prepared to calibrate the qTOF system using the AP-MALDI source. The ions with their exact masses used to calibrate the system are shown in Table S4. AP-MALDI-qTOF spectra of the calibration solution are shown in Figure S4 and S5.

Table S4: Ions used to calibrate the qTOF systems using GNP doped UP AP-MALDI target plates, with their theoretical m/z values.

Ion	m/z Theoretical
$[C_4F_9SO_3K - K]^-$	298.9430
$[C_6F_{13}SO_3K - K]^-$	398.9366
$[C_8F_{17}SO_3K - K]^-$	498.9302
$[(C_4F_9SO_3K)_2 - K]^-$	636.8491
$[C_4F_9SO_3K + C_6F_{13}SO_3K - K]^-$	736.8428
$[C_4F_9SO_3K + C_8F_{17}SO_3K – K] / [(C_6F_{13}SO_3K)_2 - K]^-$	836.8364
$[C_6F_{13}SO_3K + C_8F_{17}SO_3K - K]^-$	936.8300
$[(C_8F_{17}SO_3K)_2 - K]^-$	1036.8235
$[C_4F_9SO_3K + K]^+$	376.8693
$[C_6F_{13}SO_3K + K]^+$	476.8629
$[C_8F_{17}SO_3K + K]^+$	576.8565
$[(C_4F_9SO_3K)_2 + K]^+$	714.7755
$[C_4F_9SO_3K + C_6F_{13}SO_3K + K]^+$	814.7691
$[(C_6F_{13}SO_3K)_2 + K] / [C_4F_9SO_3K + C_8F_{17}SO_3K + K]^+$	914.7632
$[C_6F_{13}SO_3K + C_8F_{17}SO_3K + K]^+$	1014.7563
$[C_8F_{17}SO_3K + C_8F_{17}SO_3K + K]^+$	1114.7500

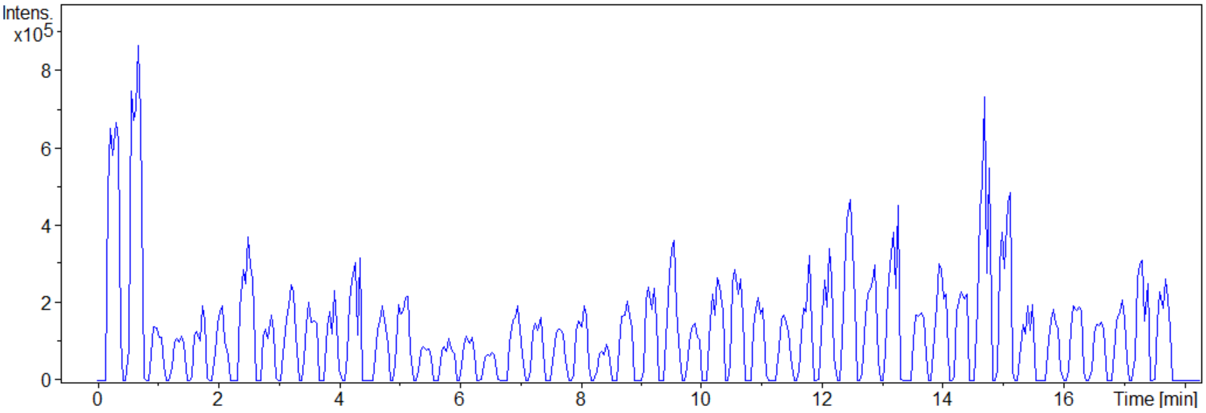

Figure S4: Total ion current plot of an automated sequence containing 48 measurements: 2 PFSA calibration mixture measurements followed by 42 plastic extract measurements (14 times 3 replicates) followed by 4 blank measurements. Analyzed using a GNP doped UP AP-MALDI target plate containing 0.5 wt.% GNPs.

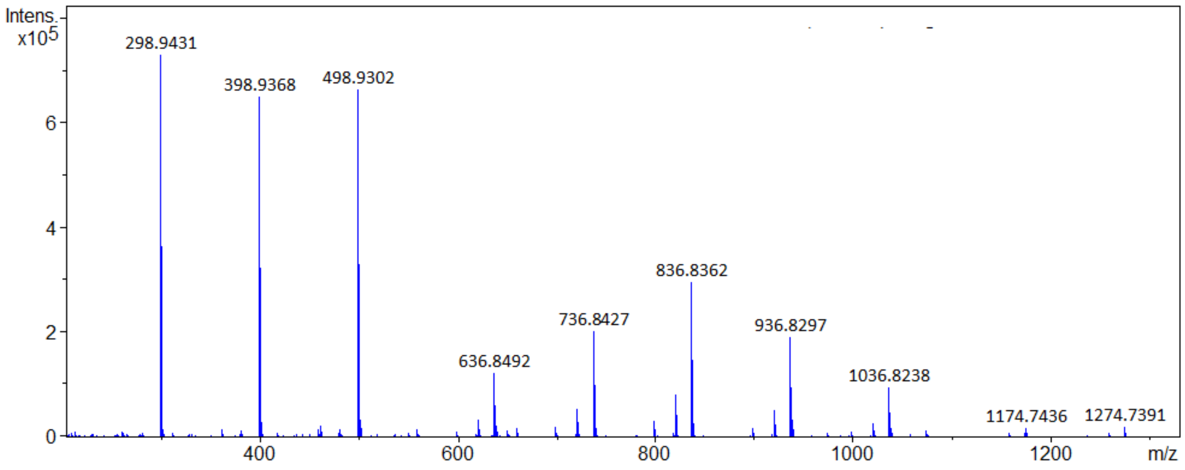

Figure S5: PFSA-mixture analysed on a GNP-doped UP AP-MALDI target plate (0.5 wt.% GNP) in negative ionisation mode.

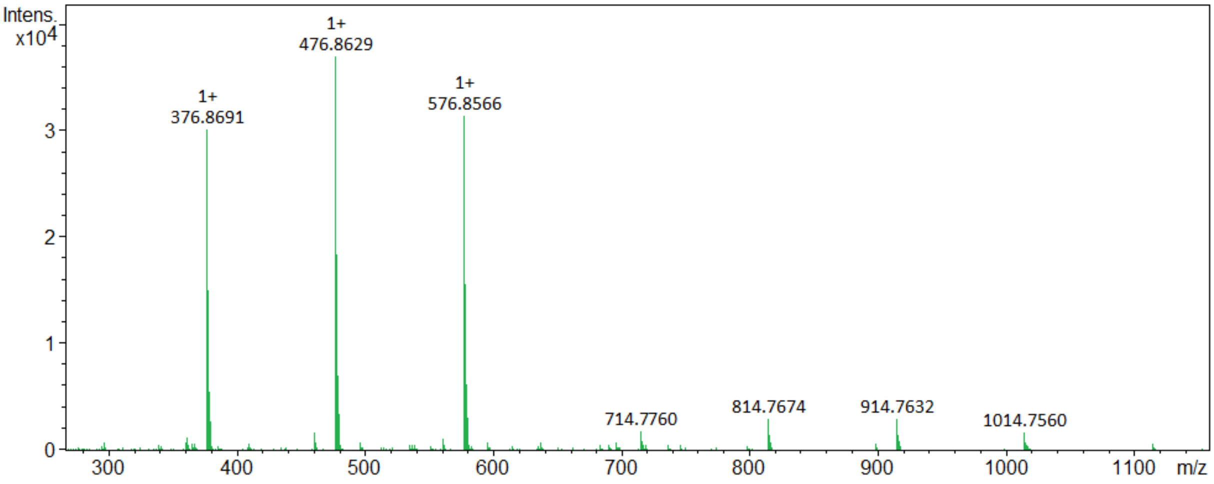

Figure S6: PFSA-mixture analysed on a GNP-doped UP AP-MALDI target plate (0.5 wt.% GNP) in positive ionisation mode.

Results 3D-printed GNP-doped AP-MALDI target plates.

Figure S7 shows the mass spectra of a standard solution mix (Table S4) analysed on 3D-printed GNP doped AP-MALDI plates containing 0.01 wt.% GNP (left) and 0.1 wt.% GNP (middle). The right spectrum is obtained with a more traditional dried droplet method on a standard stainless-steel AP-MALDI target plate for comparison. The right spectrum was obtained by transferring a droplet of 0.5 wt.% (500 mg L^{-1}) GNP suspension in acetone on the metal plate, after the acetone was evaporated a droplet of the standard mixture was pipetted on top of the GNP layer.

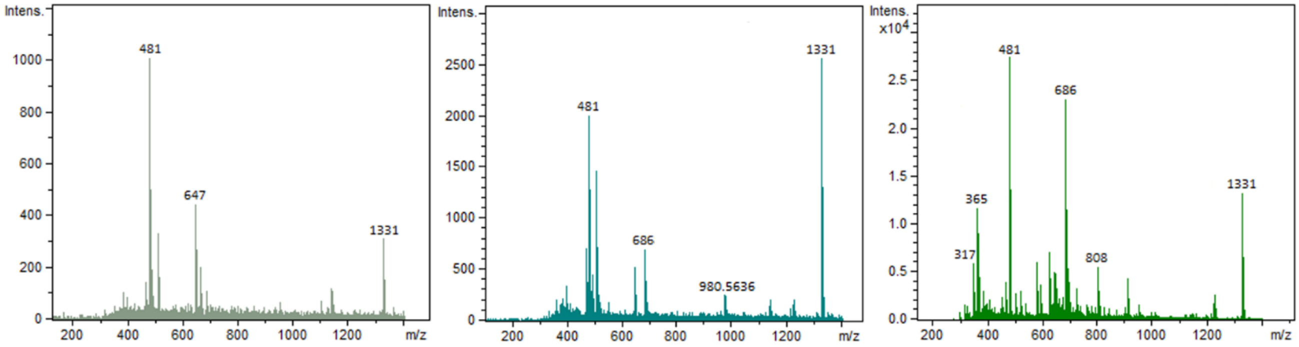

Figure S7: Left: 0.01 wt.% GNP 3D-printed target. Middle: 0.1 wt.% GNP 3D-printed target. Right: metal plate with a layer of GNP. Performance is measured on a Micro-TOF II. Positive ionisation mode analysis of a standard mixture (Table S4). m/z 317 = $[DBP+K]^+$, m/z 365= $[TPhP+K]^+$, m/z 481 = $[Tinuvin770+H]^+$, m/z 647= $[irgafos168+H]^+$, m/z 686 = $[irgafos168+O+K]^+$, m/z 1331= $[(irgafos168)_2+K]^+$.

Optimizing the GNP doped AP-MALDI targets: Cleaning the target plates.

After casting the resin target plates, a waxy residue is left on the plates. This residue hinders the laser desorption/ionization process of analytes significantly. To remove this residue, the AP-MALDI targets were cleaned by sonication for 10 minutes in toluene followed by brushing with a soft brush, this process was repeated 3 times, after which the targets where air dried in a fume hood. To illustrate the effect of properly cleaning the target plates on the AP-MALDI-TOF intensity, a standard mixture (Table S1) is analysed on a GNP doped UP target plate before and after cleaning. The results are shown in Figure S8.

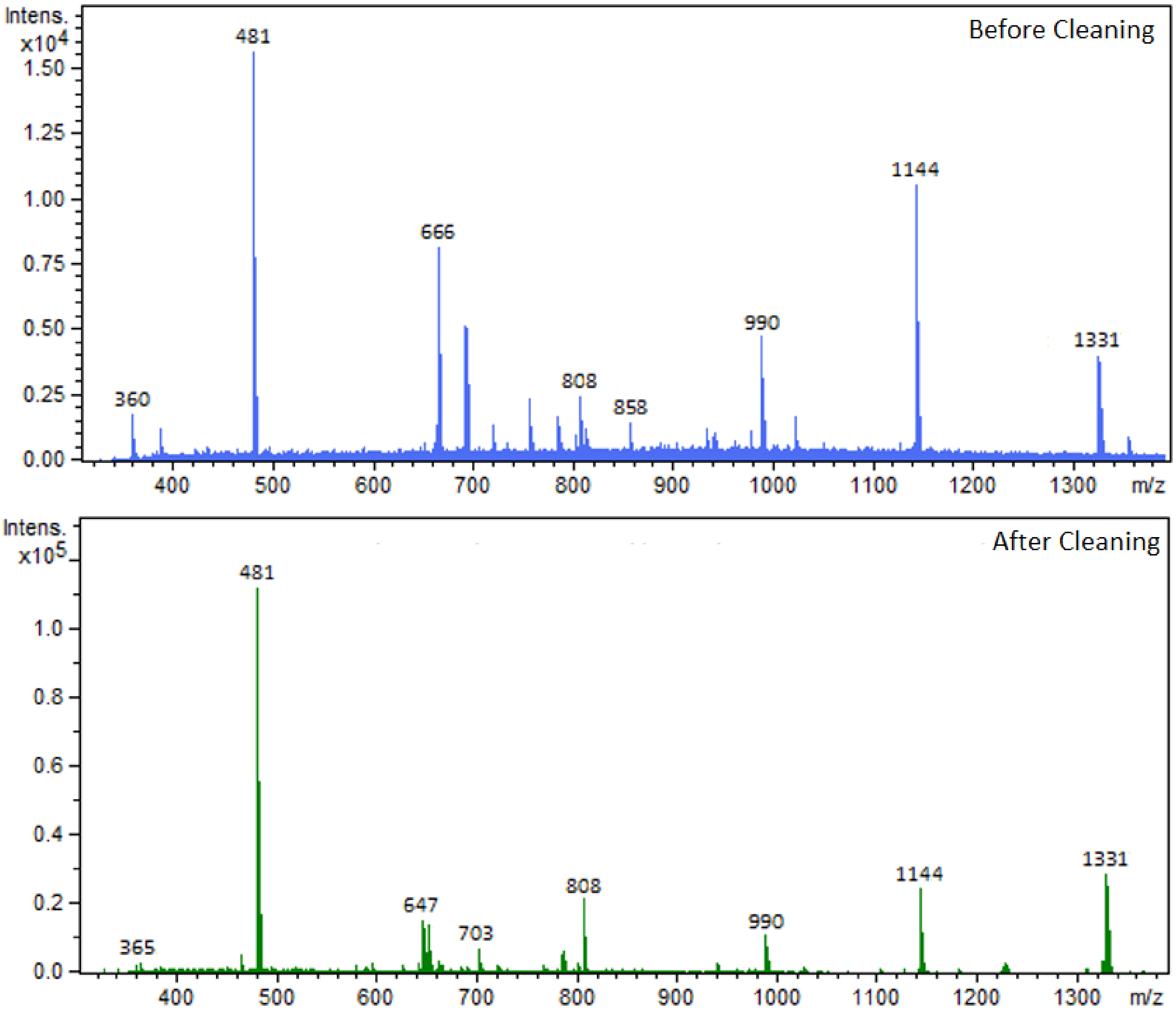

Figure S8: Performance of 0.5 wt.% GNP doped UP AP-MALDI target plate before and after cleaning. Measured on a Micro-TOF II using a standard solution mix (Table S1) (80 mg L^{-1} TBBPA, 110 mg L^{-1} TPhP, 150 mg L^{-1} DBP, 140 mg L^{-1} Irgafos 168, 140 mg L^{-1} Tinuvin 770, 140 mg L^{-1} TTBP-TAZ and 150 mg L^{-1} BPA in toluene). m/z 365= $[TPhP+K]^+$, m/z 481 = $[Tinuvin770+H]^+$, m/z 647= $[irgafos168+H]^+$, m/z 686 = $[irgafos168+O+K]^+$, m/z 1331= $[(irgafos168)_2+K]^+$.

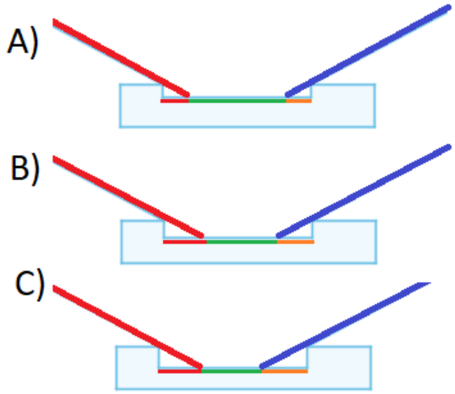

Figure S9: Schematic illustration of the influence of well-depth on AP-MALDI analysis. A) 0.3mm deep well. B) 0.4 mm deep well. C) 0.5 mm deep well. The red line = laser coming in at 28° angle. blue line = approximation of the view angle through the camera. Bottom green line = measurable and visible area. Bottom orange line = measurable but invisible area. Bottom red line: visible but unmeasurable area.

Optimization of the extraction method.

Different solvent systems were tested to ensure adequate extraction of a wide variety of compounds. Acetone was not tested in the following experiments because the GNP doped UP target plates were not compatible with acetone.

To select the optimal solvent system, three different samples were manually cut into small pieces using a scalpel. 20 mg sample material was transferred into a vial with insert in twofold, 100 µL of solvent (or solvent mixture) was added. The vials were capped and sonicated for 10 min. Hereafter 2 µL of the extract was pipetted on a GNP doped MALDI target. After air drying in the fume hood the extracts were analysed with a Micro TOF II MS (Bruker, Bremen, Germany). The sample vials were recapped, shaken overnight, and reanalysed. The results are shown in Figure S10 and Figure S11. After a 10-min extraction, good overall results were achieved with a 20:80 toluene:2-propanol mixture (Figure S10). A stronger solvent (toluene) does not yield better results due to matrix effects or polymer matrix interfering with the laser desorption/ionization.

Significantly longer extraction times did not improve AP-MALDI-TOF-MS response for all three samples. Indicating that matrix effects play a significant role. Large sample to sample variation was seen when using longer extraction times. Extracting additives from polymers is often carried out using a solvent non-solvent system. A strong solvent is added first to dissolve or swell the polymer. A weak solvent is then added to precipitate the polymer. To further increase the efficiency of the extraction method, the effect of adding solvents separately or mixed was investigated. The same three plastic samples were used as in the previous extraction experiment**.** Per sample four 20 mg aliquots are taken. Two aliquots of each sample were extracted with 100 µL toluene:2-propanol (20:80) added all at once. The other two were extracted by adding 20 µL of toluene. Subsequently, all samples were simultaneously sonicated for 10 min. Then 80 µL 2-propanol was added to the samples containing only toluene. All samples were again simultaneously sonicated for an additional 10 min. Hereafter 2 µL of each extract was pipetted on a GNP doped MALDI target. After air drying in the fume hood the extracts were analysed with a Micro TOF II MS (Bruker, Bremen, Germany).The results are shown in Figure S12. Adding toluene first led to increased AP-MALDI-TOF signals for all detected additives in all three samples.

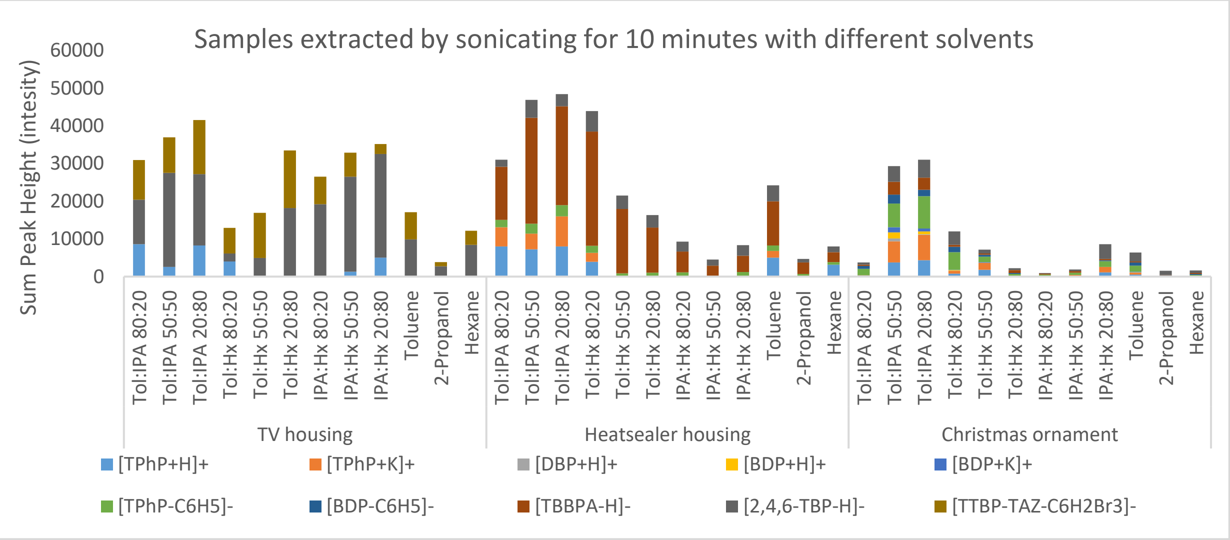

Figure S10: Results of using different solvent(systems) for the extraction of 3 plastic samples after 10 min of sonicating. Analysed on a GNP doped UP AP-MALDI target plate using AP-MALDI coupled to a micro TOF II MS.

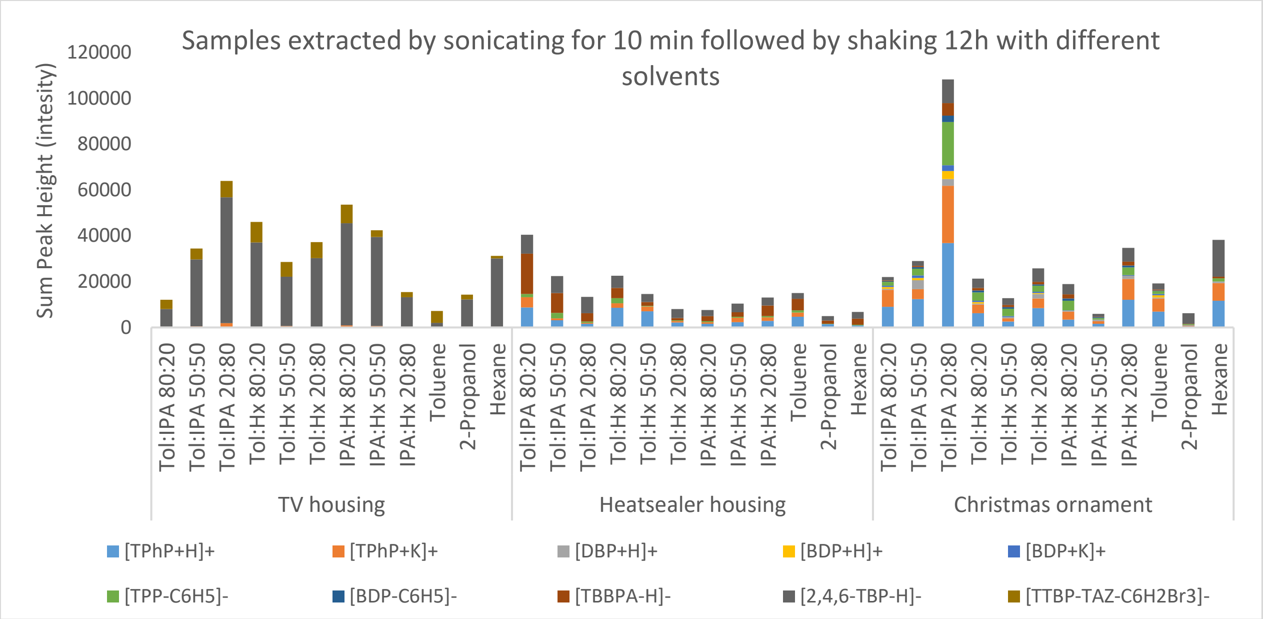

Figure S11: Effect of extracting three plastic samples for 1 night using different solvent systems on the AP-MALDI-TOF performance. Analysed on a GNP doped UP AP-MALDI target plate using AP-MALDI coupled to a micro TOF II MS.

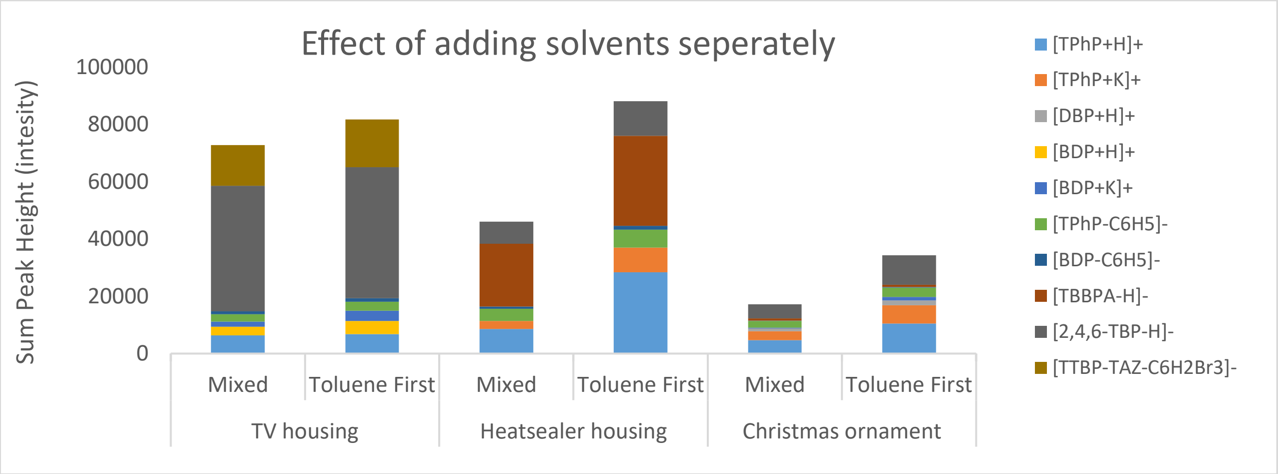

Figure S12: Effect of adding solvents separately on AP-MALDI-TOF performance. Analysed on a GNP doped UP AP-MALDI target plate using AP-MALDI coupled to a micro TOF II MS.

Table S5: Between plate reproducibility by measuring the PFSA mixture (Table S4). In Figures S5 and S6, an indication of the signal intensity of the measured ions is shown.

Ion	m/z Theoretical	Average RSD% within plates n=8	RSD% between plates n=4
$[C4F9SO3K - K]^{-}$	298.9430	14	6
$[C6F13SO3K - K]^{-}$	398.9366	11	9
$[C8F17SO3K - K]^{-}$	498.9302	9	16
$[(C4F9SO3K)2 - K]^{-}$	636.8491	23	66
$[C4F9SO3K + C6F13SO3K - K]^{-}$	736.8428	26	58
$[(C6F13SO3K)2 - K]^{-}$/ $[C4F9SO3K + C8F17SO3K - K]^{-}$	836.8364	25	54
$[C6F13SO3K + C8F17SO3K - K]^{-}$	936.8300	29	55
$[(C8F17SO3K)2 - K]^{-}$	1036.8236	39	69
**Average negative ionisation mode**		**22**	**41**
$[C4F9SO3K + K]^{+}$	376.8693	15	23
$[C6F13SO3K + K]^{+}$	476.8629	21	32
$[C8F17SO3K + K]^{+}$	576.8565	26	56
$[(C4F9SO3K)2 + K]^{+}$	714.7755	21	17
$[C4F9SO3K + C6F13SO3K + K]^{+}$	814.7690	20	14
$[(C6F13SO3K)2 + K]^{+}$/ $[C4F9SO3K + C8F17SO3K + K]^{+}$	914.7690	28	35
$[C6F13SO3K + C8F17SO3K + K]^{+}$	1014.7563	33	37
$[C8F17SO3K + C8F17SO3K + K]^{+}$	1114.7499	35	73
**Average positive ionisation mode**		**25**	**36**

Table S6: Description of the screened plastic products. Sample No. 15 was PVC CRM (KRISS 113-03-006), it is not shown in the sample tables because it is not considered a sample.

Sample No.	Description	Sample No.	Description
1	TV housing	10	Insulation material from water pipe
2	TV housing	11	Black toy cat
3	Heat sealer housing	12	TV housing
4	Christmas ornament	13	Toy excavator bottom side
5	Toy cube	14	Toy cowboy gun
6	TV housing	16	Carpet Brown
7	Toy car P	17	PVC toy flamingo
8	Power adapter	18	PVC toy inflatable ball
9	Geotextile white	19	Nylon kitchen utensil/spatula

Table S7: Tentatively identified additives in the screened products using AP-MALDI screening negative ionisation mode.

Additive type	Molecular Name	Short name	CAS	Ion detected	Sample No.
**Antioxidant**	2,2'-Methylenebis(4-methyl-6-tert-butylphenol)	Antioxidant 2246	119-47-1	$[C_{23}H_{32}O_2 - H]^-$	7, 8
	3,5-Di-tert-butyl-4-hydroxyhydocinnamic acid	Irganox 1310	20170-32-5	$[C_{17}H_{26}O_3 - H]^-$	7, 8
	Octadecyl 3-(3,5-di-tert-butyl-4-hydroxyphenyl)propionate	Irganox 1076	2082-79-3	$[C_{35}H_{62}O_3 - H]^-$	1,2, 4, 5, 6, 7, 8, 11
	Tris(2,4-di-tert-butylphenyl) phosphite	Irgafos 168	31570-04-4	$[C_{42}H_{63}O_3P + O - C_{14}H_{21}]^-$	1, 2, 3, 4, 5, 7, 9, 11, 12, 13
	4-Octylphenol/ 4-(1,1,3,3-Tetramethylbutyl)phenol/2,6-Di-tert-butylphenol	Antioxidant	1806-26-4 / 140-66-9 / 128-39-2	$[C_{14}H_{22}O - H]^-$	9
	n-Nonylphenol	NP	104-40-5	$[C_{15}H_{24}O - H]^-$	5, 7
	Methyl 3,5-bis(tert-butyl)-4-hydroxyhydrocinnamate	Antioxidant (breakdown/impurity)	6386-38-5	$[C_{18}H_{28}O_3 - H]^-$	7, 8
	3,5-bis(1,1-dimethylethyl)-4-hydroxy-Benzoic acid	Antioxidant (breakdown/impurity)	1421-49-4	$[C_{15}H_{22}O_3 - H]^-$	3, 5, 7, 8
**Flame retardant**	2,4,6-tris(2,4,6-tribromophenoxy)-1,3,5-triazine	TTBP-TAZ	25713-60-4;	$[C_{21}H_6Br_9N_3O_3 - C_6H_2Br_3 – H]^-$	1, 2
	Tribromophenol	2,4,6-TBP	118-79-6	$[C_6H_3Br_3O - H]^-$	1, 2, 4, 5, 6, 7, 11, 13, 14
	Bisphenol-A bis(diphenyl phosphate)	BDP	5945-33-5	$[C_{39}H_{34}O_8P_2 - C_6H_5]^-$	2, 3, 4, 5, 6, 7, 12
	Phenol, 2,6-dibromo-4-[1-(3-bromo-4-hydroxyphenyl)-1-methylethyl]-	TriBBPA (Breakdown/impurity of TBBPA)	6386-73-8	$[C_{15}H_{13}Br_3O_2 - H]^-$	3, 4, 5, 6
	Butyl diphenyl phosphate		2752-95-6	$[C_{16}H_{19}O_4P - H]^-$	3, 4, 5, 6, 7, 8, 11
	Resorcinol bis(diphenyl phosphate)	RDP	57583-54-7	$[C_{30}H_{24}O_8P_2 - C_6H_5]^-$	3, 4, 5, 6, 8
	3,3',5,5'-Tetrabromobisphenol A	TBBPA	79-94-7	$[C_{15}H_{12}Br_4O_2 - H]^-$	3, 4, 5, 6, 8, 11, 13, 14
	Triphenyl phosphate / diphenyl phosphate	TPhP/ DPP	115-86-6	$[C_{18}H_{15}O_4P - C_6H_5]^-$	2, 3, 4, 5, 6, 7, 8, 11, 12, 13, 14
	Chlorinated paraffins	MCCPs	85535-85-9	$[C_{15}H_{25}Cl_7 + Cl]^-$	10
**Plasticizer**	Dipentyl phthalate	DPENP	131-18-0	$[C_{18}H_{26}O_4 - H]^-$	7
**UV Stabilizer**	4H-3,1-Benzoxazin-4-one, 2,2'-(1,4-phenylene)bis-	Cyasorb UV-3638	18600-59-4	$[C_{22}H_{12}N_2O_4 - H]^-$	12
	Octabenzone	Benzophenone-12	1843-05-6	$[C_{21}H_{26}O_3 - H]^-$	7, 8, 16
	Sulisobenzone	Benzophenone-4	4065-45-6	$[C_{14}H_{12}O_6S - H]^-$	3, 8
**Others**	3,3'-[Oxybis(ethane-2,1-diyloxy)]dipropan-1-amine	Binding agent	4246-51-9	$[C_{10}H_{24}N_2O_3 - H]^-$	5, 7
	Dodecanoic acid	Lauric acid	143-07-7	$[C_{12}H_{24}O_2-H]^-$	5, 7, 9
	Octadecanoic acid	Stearic acid	57-11-4	$[C_{18}H_{36}O_2 - H]^-$	4, 5, 6, 7, 9, 11, 12
	Hexadeceanoic acid	Palmitic acid	57-10-3	$[C_{16}H_{32}O_2 – H]^-$	1, 2, 4, 5, 6, 7, 9, 11, 12, 13
	Peroxydicarbonate acid, bis(1-methylethyl) ester	catalyst	105-64-6	$[C_8H_{14}O_6-H]^-$	5, 7

Table S8: Tentatively identified additives in the screened products using AP-MALDI in positive ionisation mode.

Additive type	Molecular Name	Short name	CAS	Ion detected	Sample No.
**Antioxidant**	2-Methyl-4,6-bis[(octylthio)methyl]phenol	Antioxidant 1520	110553-27-0	$[C_{25}H_{44}OS_2 + H]^+$	5
	2,2'-Thiobis(6-tert-butyl-p-cresol)	Irganox 1081	96-69-5	$[C_{22}H_{30}O_2S + OK]^+$	7
	N,N'-(Hexane-1,6-diyl)bis(3-(3,5-di-tert-butyl-4-hydroxyphenyl)propanamide)	Antioxidant 1098	23128-74-7	$[C_{40}H_{64}N_2O_4 + OK]^+$	9
	Butylated hydroxytoluene	BHT	128-37-0	$[C_{15}H_{24}O + K]^+$	18
	Propanoic acid, 3,3'-thiobis-, didodecyl ester	DLTDP	123-28-4	$[C_{30}H_{58}O_4S +K]^+$	1
	octadecyl 3-(3 5-di-tert-butyl-4-hydroxyphenyl)propionate	Irganox 1076	2082-79-3	$[C_{35}H_{62}O_3 + K]^+$	1, 2, 4, 6, 7, 8
	2,4-Dimethyl-6-tert-butylphenol	Topanol A	1879-09-0	$[C_{12}H_{18}O + K]^+$	16
	Tris(2,4-di-tert-butylphenyl) phosphite	Irgafos 168	31570-04-4	$[C_{42}H_{63}O_3P + OK]^+$	1, 9
**Flame retardants**	2-tert-Butylphenyl diphenyl phosphate	TPhP impurity	83242-23-3	$[C_{22}H_{23}O_4P + K]^+$	6
	Phosphonic acid, methyl-, bis[(5-ethyl-2-methyl-2-oxido-1,3,2-dioxaphosphorinan-5-yl)methyl] ester		42595-45-9	$[C_{15}H_{31}O_9P_3+K]^+$	6, 8
	Bisphenol-A bis(diphenyl phosphate)	BDP	5945-33-5	$[C_{39}H_{34}O_8P_2+K]^+$	2, 3, 4, 5, 6, 7, 12
	Resorcinol bis(diphenyl phosphate)	RDP	57583-54-7	$[C_{30}H_{24}O_8P_2 + K]^+$	3, 4, 5, 6, 8
	Triphenyl phosphate	TPhP	115-86-6	$[C_{18}H_{15}O_4P + K]^+$	2, 3, 4, 5, 6, 7, 8, 12, 14
	Tris(2-butoxyethyl) phosphate	TBOEP	78-51-3	$[C_{18}H_{39}O_7P + H]^+$	11
	Tris(4-tert-butylphenyl) phosphate	TBPDPP	78-33-1	$[C_{30}H_{39}O_4P + H]^+$	9
	Tris(methylphenyl) phosphate or Tricresyl phosphate isomers	TCP or TMPP	62974-06-5	$[C_{21}H_{21}O_4P + K]^+$	6
	Tris(3,5-xylenyl)phosphate	TXP	25653-16-1	$[C_{24}H_{27}O_4P + K]^+$	6
	Tris(2-chloroethyl) phosphate	TCEP	115-96-8	$[C_6H_{12}Cl_3O_4P + K]^+$	6
**Plasticizer**	2,2,4-Trimethyl-1,3-pentanediol diisobutyrate	TXIB	6846-50-0	$[C_{16}H_{30}O_4 + K]^+$	19
	Acetyl tributyl citrate	ATBC	77-90-7	$[C_{20}H_{34}O_8 + K]^+$	4, 5, 7, 13, 18
	Butyl cyclohexyl phthalate	BCP	84-64-0	$[C_{18}H_{24}O_4 + K]^+$	5, 7, 11
	Di(propylene glycol) dibenzoate	DPGDB	27138-31-4	$[C_{20}H_{22}O_5 + K]^+$	13
	Di-n-octyl phthalate/ Di(2-ethylhexyl) phthalate	DNOP/ DEHP	117-84-0/ 117-81-7	$[C_{24}H_{38}O_4 + H]^+$	13, 17
	Di-n-octyl phthalate/ Di(2-ethylhexyl) phthalate*	DNOP/ DEHP	117-84-0/ 117-81-7	$[C_{24}H_{38}O_4 + K]^+$	2, 3, 4, 5, 6, 7, 11, 13, 17
	Diisobutyl phthalate/ Dibutyl phthalate	DBP	84-69-5	$[C_{16}H_{22}O_4 + K]^+$	4, 5, 14
	Diisodecyl phthalate/ Bis(2-propylheptyl) phthalate	DIDP/ DPHP	26761-40-0/ 53306-54-0	$[C_{28}H_{46}O_4 + K]^+$	7
	Diisonyl cyclohexane-1,2-dicarboxylate	DINCH	166412-78-8	$[C_{26}H_{48}O_4 + Na]^+$	7
	Diisononyl phthalate/ octyl decyl phthalate	DINP/ ODP	28553-12-0	$[C_{26}H_{42}O_4 + K]^+$	7, 10, 13
	Neopentyl glycol dibenzoate/ benzyl butyl phthalate	NGDB/ BBP	4196-89-8/ 85-68-7	$[C_{19}H_{20}O_4 +K]^+$	5, 7, 11
	Tributyl citrate	TBC	77-94-1	$[C_{18}H_{32}O_7 + K]^+$	18
	triethylene glycol bis(2-ethylhexanoate)	TEG-EH	94-28-0	$[C_{22}H_{42}O_6 +K]^+$	2, 4, 5, 6, 14
	Bisphenol A or benzyl benzoate	BPA	80-05-7 or 120-51-4	$[C_{15}H_{16}O_2 - CH_3]^+$ / $C_{14}H_{12}O_2 + H]^+$	5, 7, 11
**UV Stabilizer**	2-(4,6-Diphenyl-s-triazin-2-yl)-5-hexyloxyphenol	Tinuvin 1577	147315-50-2	$[C_{27}H_{27}N_3O_2 + H]^+$	18
	Bis(2,2,6,6-tetramethyl-4-piperidyl) sebacate	Tinuvin 770	52829-07-9	$[C_{28}H_{52}N_2O_4 +H]^+$	5, 7, 11, 13, 14
**Others**	4,4'-Diphenylmethane diisocyanate	ISONATE	101-68-8	$[C_{15}H_{10}N_2O_2 + H]^+$	11
	pigment black 32	Pigment	83524-75-8	$[C_{40}H_{26}N_2O_5 + K]^+$	6
